# Protection against De Novo Methylation Is Instrumental in Maintaining Parent-of-Origin Methylation Inherited from the Gametes

**DOI:** 10.1016/j.molcel.2012.07.010

**Published:** 2012-09-28

**Authors:** Charlotte Proudhon, Rachel Duffié, Sophie Ajjan, Michael Cowley, Julian Iranzo, Guillermo Carbajosa, Heba Saadeh, Michelle L. Holland, Rebecca J. Oakey, Vardhman K. Rakyan, Reiner Schulz, Déborah Bourc’his

**Affiliations:** 1 INSERM U934/CNRS UMR3215, Institut Curie, 75248 Paris Cedex 05, France; 2 Department of Medical and Molecular Genetics, King’s College London, London WC2R 2LS, England, UK; 3 Blizard Institute of Cell and Molecular Science, Queen Mary University of London, London E1 4NS, England, UK

## Abstract

Identifying loci with parental differences in DNA methylation is key to unraveling parent-of-origin phenotypes. By conducting a MeDIP-Seq screen in maternal-methylation free postimplantation mouse embryos (*Dnmt3L-/+*), we demonstrate that maternal-specific methylation exists very scarcely at midgestation. We reveal two forms of oocyte-specific methylation inheritance: limited to preimplantation, or with longer duration, *i.e*. maternally imprinted loci. Transient and imprinted maternal germline DMRs (gDMRs) are indistinguishable in gametes and preimplantation embryos, however, de novo methylation of paternal alleles at implantation delineates their fates and acts as a major leveling factor of parent-inherited differences. We characterize two new imprinted gDMRs, at the *Cdh15* and *AK008011* loci, with tissue-specific imprinting loss, again by paternal methylation gain. Protection against demethylation after fertilization has been emphasized as instrumental in maintaining parent-of-origin methylation inherited from the gametes. Here we provide evidence that protection against de novo methylation acts as an equal major pivot, at implantation and throughout life.

## Graphical abstract

**Graphical Abstract:**

**Figure:**
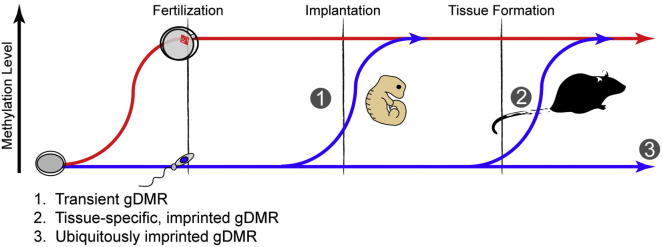

**Highlights:**

► Lifelong maintenance of parent-specific methylation marks is rare in mammals ► De novo methylation acts as a major leveling factor of parent-inherited differences ► Imprinted methylation marks can exist in a tissue-specific manner ► It is very likely that very few new imprinted loci remain to be discovered

## Introduction

Fertilization ensures the propagation of genetic and epigenetic information from one generation to the next. In mammals, epigenetic and long-lasting effects inherited in a parent-of-origin manner are known as genomic imprinting (Barlow, 2011). The main epigenetic mark that ensures their transmission and effects is DNA methylation. Methylation marks at imprinted loci are established in a sex-specific manner during gametogenesis, at genomic loci referred to as germline differentially methylated regions (gDMRs). After fertilization, these gDMRs act in *cis* to control the monoallelic and parent-specific expression of a subset of genes, the imprinted genes. Germline DMRs can affect imprinted expression in a variety of ways, including promoter control of protein-coding and noncoding RNAs, regulation of transcription elongation, and long distance insulator activities. The allelic differences of gDMRs also include a typical chromatin signature, consisting of both repressive and permissive histone marks (McEwen and Ferguson-Smith, 2010). Disruption of imprinted expression upon genetic deletion of a gDMR is ultimate proof that it functions as an imprinting control region (ICR).

To date, around 120 imprinted genes have been identified in mouse and human. They are under the control of 20 identified gDMRs/ICRs, 17 of which are methylated in the oocyte (maternal gDMRs), and only three in sperm (paternal gDMRs). Parental ICRs are also sexually dimorphic in terms of CpG content and genomic localization: maternal ICRs are CpG island (CGI) promoters, while paternal ICRs are relatively CpG poor and intergenic. Evolutionary reasons for these discrepancies may be linked to the different developmental kinetics of male and female gametogenesis (Bourc’his and Bestor, 2006; Schulz et al., 2010). Importantly, maternal ICRs have a dominant role in early development, regulating biological pathways related to the establishment of the feto-maternal interface (Schulz et al., 2010).

It is becoming increasingly clear that the acquisition of sex-specific methylation patterns extends beyond imprinted regions in gametes (Kobayashi et al., 2012a; Smallwood et al., 2011; Smith et al., 2012). CpG islands are more prone to being methylated in the oocyte than in sperm and globally, about a thousand CGIs may be specifically methylated in the oocyte genome, exceeding the number of known maternal ICRs by far. Imprinted and nonimprinted methylation is likely to be established in the same way in the oocyte, under the control of the de novo DNA methyltransferase DNMT3A and its cofactor DNMT3L, and in a transcription-dependent manner (Bourc’his et al., 2001; Chotalia et al., 2009; Kaneda et al., 2004; Kobayashi et al., 2012a; Smallwood et al., 2011).

Instead of specific targeting mechanisms for their establishment in gametes, what truly distinguishes ICRs from the rest of the genome is their treatment after fertilization, in the wake of the extensive methylation changes inherent to early mammalian development. Before implantation, methylated alleles of ICRs are resistant to the genome-wide erasure of gametic methylation that coincides with the acquisition of embryonic pluripotency. Specific *trans* acting factors have been identified as critical in maintaining ICR methylation during this period, such as binding of the KRAB (Krüppel-associated box-containing) zinc finger protein system, which involves Zfp57 and the heterochromatin inducer KAP1/TRIM28 (Li et al., 2008; Mackay et al., 2008; Quenneville et al., 2011; Zuo et al., 2012). Following this critical period, parental differences in ICR methylation are thought to persist throughout life, with no stage- and tissue-specificity, although this aspect of imprinting has not been fully addressed.

To gain insight into the extent of gametic methylation inheritance, we performed a genome-wide screen for gDMRs in the mouse postimplantation embryo. Considering their larger number, we specifically looked for maternally transmitted gDMRs, by comparative methylation profiling of wild-type embryos and embryos lacking oocyte-inherited methylation, using MeDIP-Seq (Methylated DNA Immunoprecipitation followed by high throughput sequencing). We exploited the well-characterized *Dnmt3L* mutant system, in which postimplantation *Dnmt3L-/+* embryos generated by fertilization of *Dnmt3L−/−* oocytes completely lack maternal imprints, while methylation patterns at paternal ICRs and repeats are unaltered (Bourc’his et al., 2001; Schulz et al., 2010).

Our approach proved to be highly sensitive and efficient at identifying regions of oocyte-inherited methylation. We found all 17 known maternal gDMRs/ICRs and less than thirty new candidates, revealing that loci that maintain maternal-specific methylation are rare in the postimplantation embryo. We characterized two new maternally imprinted gDMRs, located within the *Cdh15* gene and at the promoter of *AK008011*, a retroposed pseudogene. Further analyses enabled us to demonstrate that inherited maternal gDMRs can exist in a permanent or transient state after fertilization, and that avoidance of de novo methylation during embryo or tissue differentiation plays a key role in the permanency of parent-of-origin methylation inherited from the gametes.

## Results

### A Genome-wide Screen for Regions of Oocyte-Inherited Methylation by MeDIP-Seq

We generated DNA methylation profiles by MeDIP-Seq for pools of 8.5 dpc (days post coïtum) embryos of either a wild-type or *Dnmt3L-/+* genotype. For each pool, two independent MeDIP preparations were sequenced. We obtained 41 M and 32.7 M distinct and uniquely alignable reads for wild-type and *Dnmt3L-/+* embryos, respectively (Supplemental Information). For a 1000 bp sliding window size and a permissive false discovery rate (FDR) threshold of < 50%, 398 differentially methylated regions (DMRs) were identified genome-wide, 163 of which were hypomethylated and 235 hypermethylated in *Dnmt3L-/+* embryos (Table S1). This suggests that overall, wild-type and *Dnmt3L-/+* postimplantation embryos have similar methylation profiles and differ only at a small set of discrete loci.

**Figure 1:**
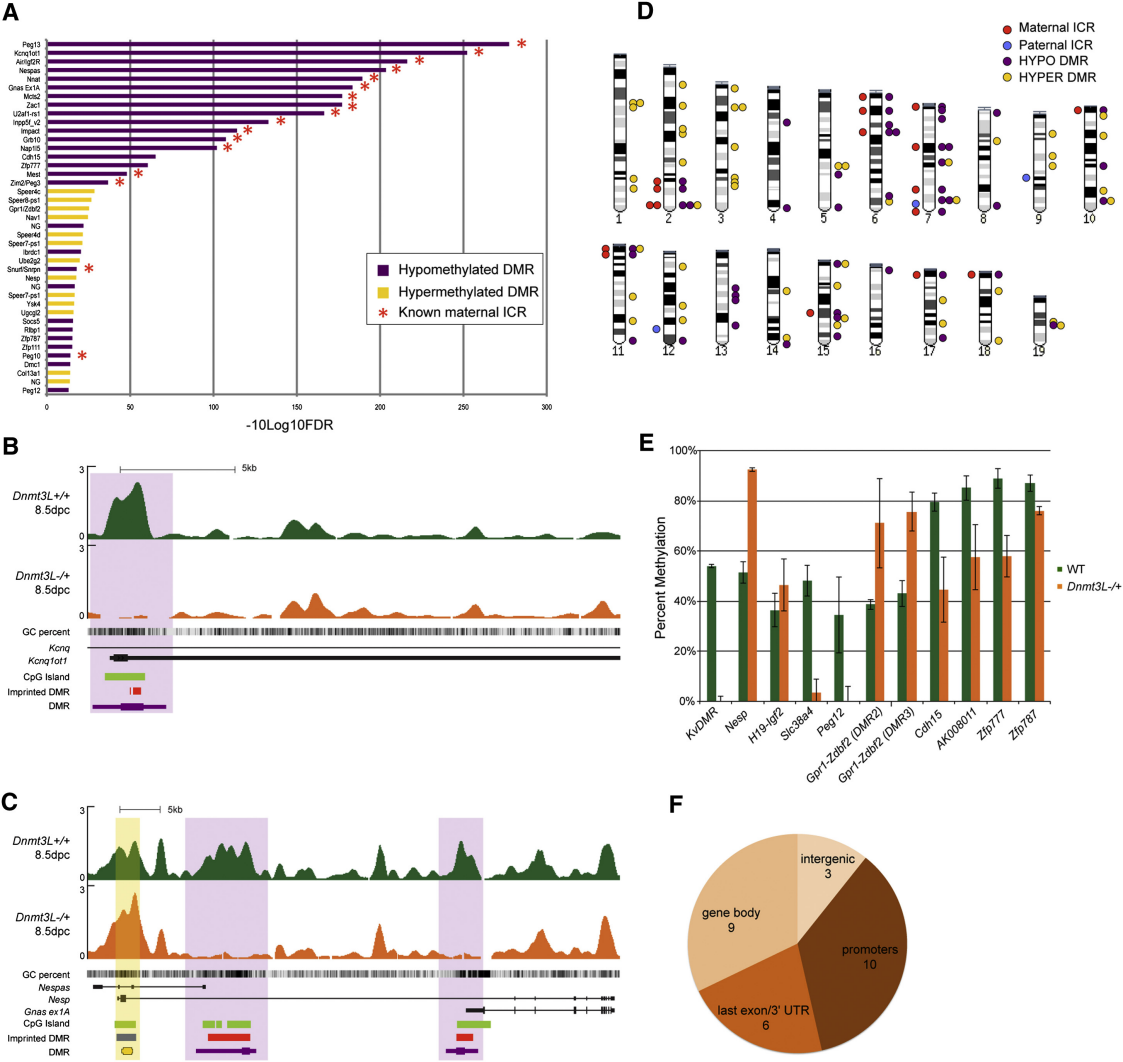
MeDIP-Seq Screen for the Identification of Oocyte-Dependent DMRs, Using 8.5 dpc WT and *Dnmt3L-/+* Embryos (A) DMRs with an FDR of up to 5% are ranked from top to bottom in order of statistical significance (−10log_10_(FDR)). The 17 known maternal ICRs are labeled with red asterisks. NG means “no gene,” according to UCSC annotation. (B) MeDIP-Seq profile of the *Kcnq1ot1* locus controlled by KvDMR, a known maternal ICR (red). The tracks depict the MeDIP-Seq profiles of 8.5 dpc WT embryos and *Dnmt3L-/+* embryos, which are highly similar except for a the hypomethylated KvDMR (purple). Genes are oriented 5′ to 3′, and the *y* axis scale expresses the number of fragments per million mapped fragments. (C) MeDIP-Seq profile of the *Gnas* locus, controlled by two known maternal ICRs, which are hypomethylated (purple). This locus also contains a secondary somatic DMR, hypermethylated (yellow) in *Dnmt3L-/+* embryos. (D) Mouse karyotype with the positions of 47 hypomethylated and 48 hypermethylated candidate DMRs in *Dnmt3L-/+* embryos, and the previously known ICRs. (E) MSRE-qPCR validation of methylation. Error bars show the standard devitation from three independent digestions. (F) Transcript position of 28 hypomethylated DMRs, which represent new potential maternal gDMRs.

The relatively small number of DMRs between wild-type and *Dnmt3L-/+* embryos was not due to a lack of sensitivity of our MeDIP-Seq approach, as all of the 17 known maternal ICRs were identified at an FDR threshold of only 5%. Furthermore, when ranked by confidence score (−10log_10_ of FDR), the top 13 ranks were occupied by known maternal ICRs (Figure 1A). The highest level of significance was obtained for the *Peg13* ICR (FDR < 10^−27^), then for the KvDMR ICR (FDR < 10^−25^), which regulates the promoter of the *Kcnq1ot1* noncoding RNA, and the lowest for the *Peg10/Sgce* ICR (FDR < 0.04) (Figures 1B, S1A, and S1B). Of note, the *Peg3* and *Snrpn* ICRs, which are prone to regaining methylation in some *Dnmt3L-/+* progeny (Arnaud et al., 2006), were identified as highly significant hypomethylated DMRs (FDR < 0.02) (Figures S1C and S1D).

Unexpectedly, our screen uncovered hypermethylated DMRs, which gain methylation in *Dnmt3L-/+* embryos. Among them, we found known secondary imprinted DMRs, which acquire methylation in somatic tissues as a consequence of maternal ICR control. For example, the *Gnas* locus contains two maternal ICRs, encompassing the *Gnas ex1A* (FDR < 10^−18^) and the *Nespas* promoters (FDR < 10^−20^) (Figure 1C). Paternal *Nespas* transcription induces the paternal methylation in *cis* of a secondary, somatically acquired DMR at the *Nesp* promoter. In the absence of maternal germline methylation, *Nespas* expression becomes biallelic and *Nesp* methylation occurs on both alleles (Liu et al., 2005). Our MeDIP-Seq approach unambiguously detected hypermethylation at the *Nesp* secondary DMR in *Dnmt3L-/+* embryos (FDR < 0.02) (Figure 1C). Our MeDIP-Seq screen also proved to be highly specific: the three genetically confirmed paternal ICRs (*H19-Igf2*, *Dlk1-Gtl2* and *Rasgrf1*) that acquire methylation in the male germline were not identified as DMRs (Figure S2A). In addition, sequences that acquire methylation specifically in the embryo, such as CGI promoters of germline expressed genes (Borgel et al., 2010), also showed similar profiles between wild-type and *Dnmt3L-/+* embryos (Figure S2B). In summary, evidence from known positive and negative controls demonstrates that our MeDIP-Seq screen accurately identified regions of oocyte-dependent methylation in the embryo.

To prioritize our candidate DMRs, we applied certain stringency filters, based on systematic genomic features of known maternal ICRs. Sequences that acquire methylation in oocytes tend to be CG-rich and among them, maternal ICRs have an observed to expected CpG ratio > 0.5 (Schulz et al., 2010). Given this fact and due to the functional link between CG density and DNA methylation-mediated transcriptional control (Weber et al., 2007), we excluded DMR candidates that contained fewer than 10 CpGs and that had a ratio of < 0.213 (the median across all identified DMRs). Additionally, we found that all known maternal ICRs and their associated secondary somatic DMRs have < 25% repeat sequence content. Given this relatively repeat-free nature and the difficulty of accurately assigning the genomic origin of short sequencing reads that originate from repeats, we further excluded candidates with a repeat content > 25%. These filters reduced the DMR number to 96, 47 hypomethylated and 49 hypermethylated, which showed a dispersed distribution throughout the mouse genome (Figure 1D).

### Improved Definition of Known Imprinted Loci and Identification of New gDMRs

We first used our MeDIP-Seq data to improve the genetic map of germline or secondary DMRs for known imprinted loci that have not been fully documented. Two hypomethylated DMRs coincided with the promoters of the *Slc38a4* (FDR < 0.5) and *Peg12* (FDR < 0.05) genes (Figures S1E and S1F), and were confirmed to be hypomethylated in *Dnmt3L-/+* embryos by MSRE-qPCR assays (Methylation-Sensitive Restriction Enzyme coupled with quantitative PCR) (Figure 1E). Paternal-specific expression and maternal-specific methylation had been previously reported at these loci in somatic tissues (Kobayashi et al., 2002; Smith et al., 2003). Reduced-Representation Bisulfite Sequencing (RRBS) confirmed here that the *Slc38a4* DMR is indeed methylated in oocyte and hypomethylated in sperm and can be categorized as a genuine maternal gDMR (Figure S3A). However, the *Peg12* DMR was unmethylated in both oocyte and sperm, suggesting that it is not a gDMR. Our screen also led to a reassessment of the imprinted *Gpr1-Zdbf2* locus, which was originally characterized as a fourth imprinted region controlled by paternal methylation (Hiura et al., 2010; Kobayashi et al., 2009). We identified two hypermethylated DMRs (FDR < 0.01 and 0.07), which overlap with two originally described paternal gDMRs (DMR2 and DMR3) (Figures 1A and S1G). However, as discussed earlier, paternal gDMRs, such as the *H19-Igf2* DMR, are unaltered in our screen. The methylation status of the *Gpr1-Zdbf2* DMRs in *Dnmt3L-/+* embryos is instead reminiscent of a secondary, somatic DMR similar to the *Nesp* DMR, as validated by MSRE-qPCR (Figure 1E) and bisulfite sequencing (data not shown), and was recently independently confirmed (Kobayashi et al., 2012b).

In our search for additional novel maternal gDMRs, we focused on 28 hypomethylated, single-copy and relatively CpG-rich candidate DMRs (Table 1), which importantly do not belong to known imprinted regions. All but three of the candidate gDMRs were within a transcription unit, among which ten were located to an annotated promoter, six overlapped with the last exon/3′ UTR, and nine were within a gene body (Table S1 and Figure 1F). Contrary to known maternal ICRs, all of which coincide with promoter-associated CGIs, only nine out of 28 candidates overlapped with a CGI and only five of those were associated with an annotated promoter. To evaluate which candidate gDMRs may constitute regions of bona fide oocyte-specific methylation, we interrogated publically available CGI methylation data in the mouse oocyte (Kobayashi et al., 2012a): all but two CGI candidates from our screen were found methylated. We further integrated MeDIP-Seq data from mouse C57Bl6/J sperm (Table 1), and found only one of the candidates to be methylated in sperm, confirming that sequences methylated in the oocyte are usually not methylated in sperm (Kobayashi et al., 2012a; Smallwood et al., 2011; Smith et al., 2012). As an indication of long-term maintenance of maternal-specific methylation, we integrated MeDIP-Seq data from fetal 17.5 dpc hybrid mouse liver from C57Bl6/J and PWD/PhJ strain crosses. In these samples, parental allele-specific sequencing reads were counted at known SNPs between the parental strains (Supplemental Information). For six candidate gDMRs, we found evidence for maternal-specific methylation maintenance (binomial p < 0.2) (Table 1). Of note, the lack of evidence for maternal-specific methylation from liver was mostly due to a paucity of SNPs (7/28 DMRs) or low read depth over an existing SNP (14/28 DMRs). Using bisulfite sequencing, we further assessed four of these candidates. None of these showed maternal-specific methylation in fetal liver (Figure S4), confirming that very few new maternal gDMRs persisting after implantation are left to be uncovered.

We went on to study four candidate maternal gDMRs in more detail, chosen for their high level of significance in our screen and their association with a CGI, a systematic feature of currently known maternal ICRs. Importantly, MSRE-qPCR and bisulfite sequencing confimed their hypomethylation in 8.5 dpc *Dnmt3L-/+* embryos and in sperm (Figure 1E and data not shown). Three are located in gene bodies toward the 3′ end of the respective canonical RefSeq transcript (*Cdh15, Zfp777* and *Zfp787*). The fourth candidate overlaps with the promoter of *AK008011*, a mono-exonic retrogene.

### The *Cdh15* DMR Controls the Paternal- and Tissue-Specific Expression of an Intragenic Transcript

**Figure 2:**
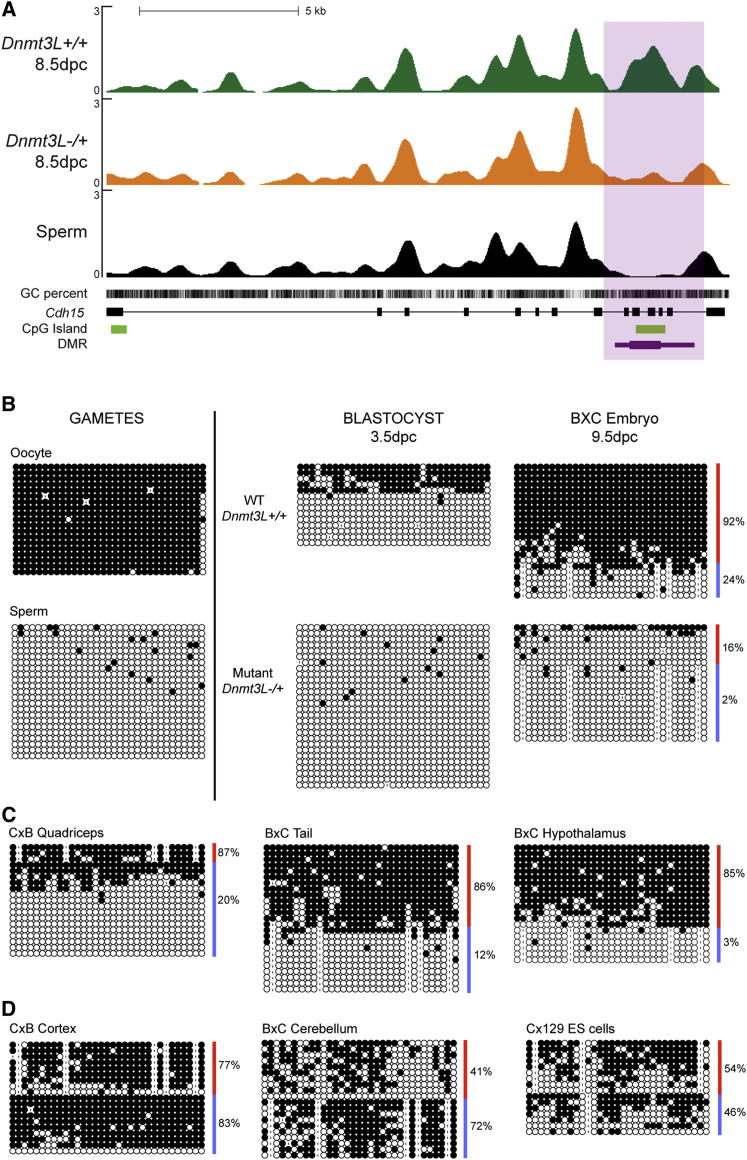
Identification and Methylation Analysis of the *Cdh15* DMR (A) MeDIP-Seq profile of the *Cdh15* locus. Genes are oriented 5′ to 3′. The MeDIP-Seq tracks show an intragenic CGI with hypomethylation in *Dnmt3L-/+* embryos and sperm compared to WT embryos (thick part of purple bar: highest statistical confidence). (B) Developmental analysis of *Cdh15* DMR methylation by bisulfite sequencing. Red and blue lines delineate maternal and paternal alleles. (C) Maternal-specific methylation is maintained in various hybrid adult tissues. (D) The parental specificity of the DMR is lost in cortex, cerebellum and ES cells, by methylation acquisition on paternal alleles (blue). Black circle: methylated CpG, white circle: unmethylated CpG, dash: absent CpG corresponding to strain-specific SNPs or, rarely, sequencing errors. Mouse strains: B = C57Bl6/J, C = CAST/Ei, 129 = 129 Sv.

The *Cdh15* DMR (ranked 14^th^; FDR < 0.02) spans exons 10 to 12 of the *Cdh15* gene (Figures 1A and 2A), which maps to distal chromosome 8 (8qE2) and encodes the M-cadherin protein, a cell-adhesion protein linked to muscle and cerebellum (Padilla et al., 1998; Rose et al., 1995). By bisulfite sequencing of exon 11, we showed that this DMR fulfills the three developmental criteria of a maternally imprinted gDMR (Figures 2B and S3B): (1) methylation acquisition in the oocyte but not in sperm, (2) maintenance of maternally methylated alleles prior to implantation, as revealed by the lack of methylated alleles in maternal-imprint free *Dnmt3L-/+* blastocysts compared to wild-type blastocysts, and (3) protection of the paternally unmethylated alleles after implantation, as shown in 9.5 dpc embryos derived from C57Bl6/J and CAST/Ei strains. Moreover, we demonstrated that the maternal allele is unable to regain methylation in *Dnmt3L-/+* postimplantation embryos, confirming the obligate passage through the female germline to imprint this locus.

In adult tissues and cells derived from C57Bl6/J by CAST/Ei or 129 Sv by CAST/Ei crosses, the methylated status of maternal alleles was consistently maintained. In quadriceps, tail and hypothalamus, methylation differences between parental alleles were highly significant (Fisher’s exact p < 10^−11^), although a minority of paternal alleles tended to regain methylation in quadriceps (Figure 2C). MeDIPSeq analysis of fetal hybrid liver DNA also showed higher methylation of maternal compared to paternal alleles (binomial p = 0.133) (Table 1). However, in ES cells, adult cortex and cerebellum, parental specificity was lost due to acquisition of methylation on the paternal alleles (Figure 2D). The intragenic *Cdh15* DMR is therefore conserved during adulthood, but in a tissue-specific manner.

**Figure 3:**
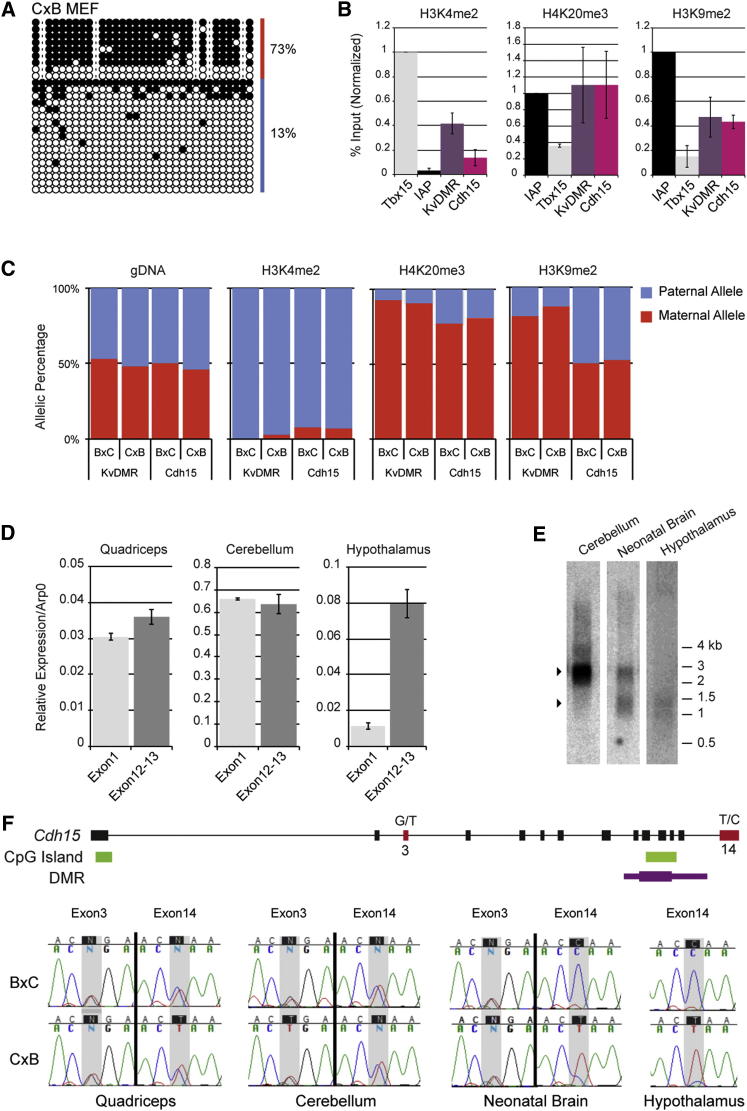
Chromatin and Expression Analysis at the *Cdh15* Locus (A and B) In MEFs, the *Cdh15* DMR (A) globally maintains maternal-specific DNA methylation, (B) shows combined enrichment of permissive and repressive histone marks. Relative enrichments were calculated from ChIP-qPCR experiments as Input %, and normalized to *Tbx15* promoter for H3K4me2, and to IAP 5′LTR for H4K20me3 and H3K9me2. Error bars show the standard deviation from three biological replicates. (C) Permissive and repressive marks show opposite allelic enrichment by ChIP-pyrosequencing, on reciprocal BxC and CxB MEFs. Genomic DNA (gDNA) was used to exclude assay-specific biases. (D) RT-qPCR assay shows equal measurement of expression upstream (exon 1) and downstream (exons 12–13) of the DMR in quadriceps and cerebellum. A 10-fold higher expression is detected downstream in the hypothalamus. (E) Northern blot analysis identifies a full 3 kb transcript in cerebellum, a shorter 1–1.5 kb transcript in adult hypothalamus, and both forms in neonatal brain. (F) RT-PCR sequencing tracks of the allelic expression status of the main and short *Cdh15* transcripts. SNP nucleotides (red) are indicated in the B then C order.

We next investigated the chromatin state of the *Cdh15* DMR by immunoprecipitation (ChIP). We measured the quantity and allelic specificity of three marks associated with imprinted DMRs (H3K4me2, H3K9me2 and H4K20me3), in MEFs (mouse embryonic fibroblasts), which globally maintain maternal-specific DNA methylation (Figure 3A). The *Cdh15* DMR was found enriched in repressive H3K9me2 and H4K20me3, at a level similar to the typical maternal ICR KvDMR (Figure 3B). Permissive H3K4me2 marks were also found at this locus. We assayed the allele-specificity of these marks by pyrosequencing, exploiting SNPs between the C57Bl6/J and CAST/Ei strains (Figure 3C). H3K4me2 was associated with the paternal allele, while H4K20me3 was enriched on the maternal allele. In contrast to KvDMR, for which H3K9me2 is maternally enriched, this mark was equally distributed on both parental alleles at the *Cdh15* DMR. This shows that the *Cdh15* DMR harbors opposite allelic states of histone modifications in MEFs, with respect to H3K4 and H4K20 methylation.

*Cdh15* is highly expressed in satellite cells of skeletal muscles and granular cells of the cerebellum (Cornelison and Wold, 1997; Rose et al., 1995). Moreover, evidence for paternal-specific expression was recently reported in adult hypothalamus (Gregg et al., 2010). By using quantitative RT-PCR, we readily detected *Cdh15* expression in quadriceps, cerebellum and hypothalamus, with the strongest detection in cerebellum (Figure 3D). Expression measurements were equal upstream and downstream of the DMR (exon 1 versus exons 12–13) for the quadriceps and the cerebellum, suggesting the existence of a transcript elongating from the 5′ canonical promoter throughout the coding unit. However, in the hypothalamus, 10-fold greater expression was measured downstream of the DMR, suggesting the existence of a transcript originating intragenically. Northern blot analysis confirmed the production of a single full-length transcript in the cerebellum, around the expected 3 kb size, while the hypothalamus specifically expressed a shorter version of *Cdh15,* which could be detected with a probe spanning exons 9–14 (Figure 3E), but not exons 5–9 (data not shown). Both transcripts were present in neonatal brains.

The allelic status of *Cdh15* expression was determined in reciprocal BxC and CxB crosses: while biallelic expression was found upstream and downstream of the DMR in quadriceps and cerebellum, only paternal expression of the short *Cdh15* transcript was detected in hypothalamus (Figure 3F). In neonatal brains, a switch from biallelic to monoallelic expression was observed at the DMR. Further allelic mapping by RT-PCR revealed that the short paternal transcript originates between exons 9 and 10, 5′ of the *Cdh15* DMR, a region that showed maternal-specific methylation in neonatal brain (Figures S5A and S5B). The *Cdh15* DMR probably corresponds to an intragenic promoter, specifically active in brain cell-types detectable at birth and in the hypothalamus at adulthood. Its differential methylation correlates with differential allelic transcription in these cell types.

The *Cdh15* DMR defines chromosome 8 as a new chromosome harboring an imprinted locus. To determine the extent of *Cdh15* DMR control, we measured the allelic expression of the three closest neighboring genes (*Acsf3*, *AK040202* and *Ankrd11*), by an RT-PCR pyrosequencing-based approach. We did not detect imprinted expression for these genes, in a bank of reciprocal hybrid tissues including embryonic, fetal, neonate and adult stages (Figure S6). It is therefore highly probable that the *Cdh15* DMR does not control an imprinted cluster.

Of clinical interest, the human *CDH15* gene has been associated with intellectual disability (Bhalla et al., 2008). The *CDH15* gene has a similar genomic organization to its mouse homolog, notably with an intragenic CGI spanning exons 9 to 12. We analyzed the imprinted status of this locus in human fetal liver, a tissue we find to maintain maternal-specific methylation in mouse. Unexpectedly, the 5′ part of the CGI was completely methylated, while the 3′ part that includes the region homologous to the sequence we analyzed in mouse was completely unmethylated (Figure S5C). Lack of methylation was confirmed in lymphocyte and placental DNA (data not shown). Our study does not support a conservation of imprinting for the *CDH15* locus in humans but rather points to a bipartite CGI.

### The *AK008011* DMR Is a Tissue-Specific Imprinted gDMR at a Mouse Pseudogene

**Figure 4:**
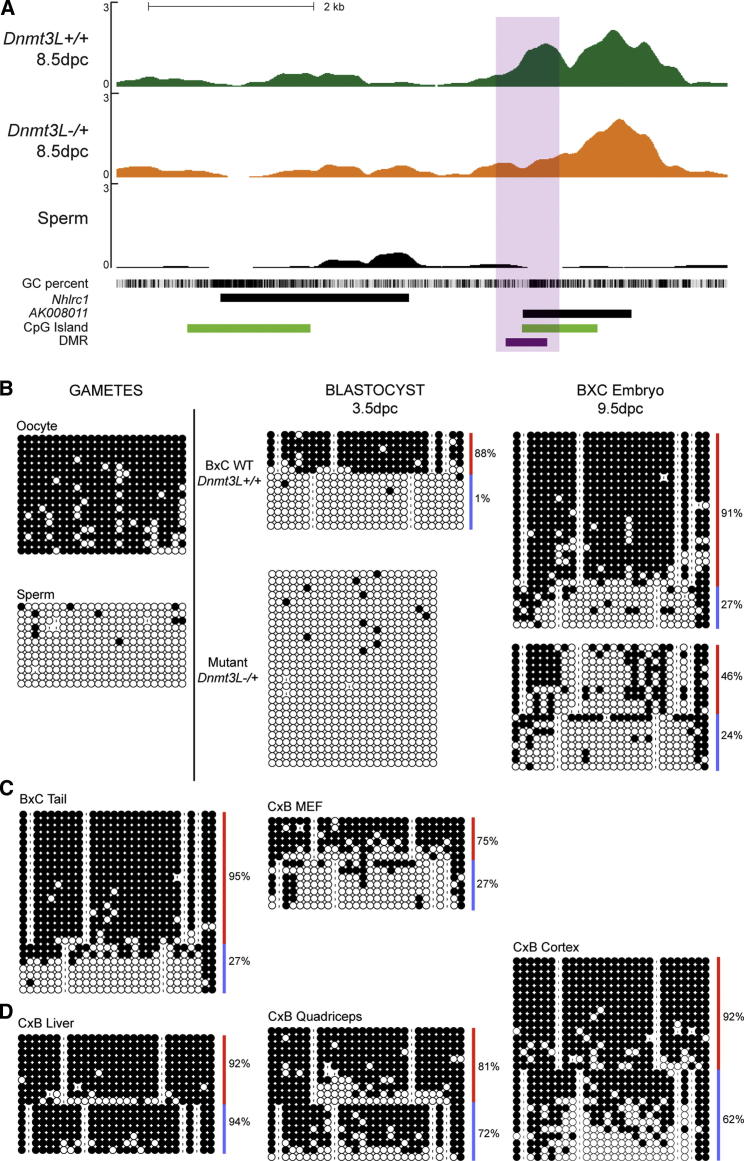
Identification and Methylation Analysis of the *AK008011* DMR (A) MeDIP-Seq profile of the *AK008011* locus. (B) Developmental analysis of DNA methylation of this locus by bisulfite sequencing. (C) Maternal-specific methylation is maintained in tail and MEFs. (D) The parental specificity is lost in liver, quadriceps and cortex, by methylation acquisition on paternal alleles (blue).

The second DMR we focused on maps to a CGI located 5′ of *AK008011*, an intronless gene (Figure 4A). It was likely generated via the retrotransposition of a *Coro1c* mRNA (Coronin, Actin binding protein 1c located on chromosome 5) to a region 1.5 kb downstream of *Nhlrc1* on chromosome 13qA5, an event that occurred specifically in the mouse lineage (Kent et al., 2003). We uncovered this small DMR through a 500 bp sliding window analysis (FDR < 0.35) (Table S1), while no DMR was identified at the *Coro1c* locus. Bisulfite-based methylation analysis revealed *1)* methylation acquisition in oocyte but not in sperm, *2)* protection of maternally methylated alleles prior to implantation and *3)* protection of paternally unmethylated alleles after implantation (Figure 4B). This locus therefore behaves as a typical maternal imprinted gDMR during the critical window around fertilization and implantation. However, in adult life, this gDMR becomes tissue-specific. While maternal-specific methylation is properly maintained in tail and fibroblasts (Figure 4C), the quadriceps, cortex and liver show dense methylation (over 60%) of both paternal and maternal alleles (Figure 4D). This finding again questions the view of the permanency of imprinted gDMRs throughout life.

In an attempt to investigate the impact of this gDMR on allelic expression, we designed primers that specifically distinguish *AK008011* from *Coro1c* mRNA. However, we could not detect expression in tissues where the gDMR is conserved. The high rate of nucleotide divergence between mouse strains suggests a low selective pressure on this gene, which may be a pseudogene: 40 SNPs are referenced at *AK008011*, including 9 nonsynonymous ones, versus 6 synonymous changes at the transcribed region of *Coro1c* (MGI and dbSNP build 128*).* Further examination of the closest gene, *Nhlrc1*, did not reveal a bias in parental expression in any tissue from our hybrid bank (data not shown), suggesting that the *AK008011* gDMR does not have long-range imprinting effects.

### *Zfp777* and *Zfp787* DMRs Are Transient Maternal gDMRs

**Figure 5:**
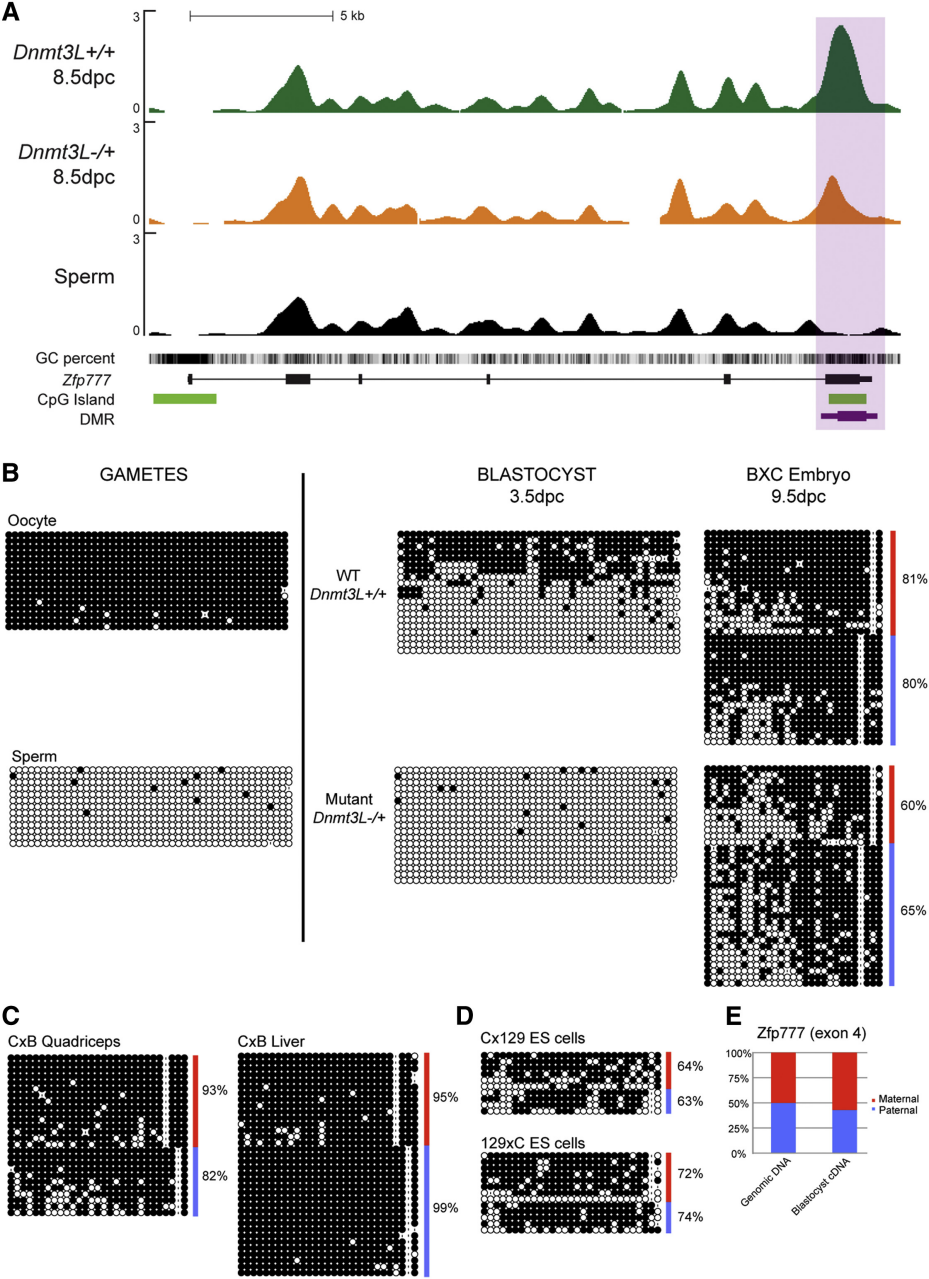
Identification and Methylation Analysis of the *Zfp777* DMR (A) MeDIP-Seq profile of the *Zfp777* locus. (B) Contrary to imprinted gDMRs, DNA methylation is gained on paternal alleles at implantation and parental alleles exhibit similar methylation levels both in WT and *Dnmt3L-/+* 9.5 dpc embryos. (C) Adult tissues show a fully methylated pattern. (D) Parental alleles are similarly methylated in ES cells. (E) RT-PCR pyrosequencing analysis of a SNP located in the 3′UTR shows biallelic expression of *Zfp777* in hybrid blastocysts at 3.5 dpc.

The last two hypomethylated DMRs we validated (FDR < 2%) map to CGIs located in the last exon of the *Zfp777* (6qB2.3) and *Zfp787* (7qA1) genes, which encode zinc finger proteins (Figures 5A and S7A). As is typical for maternal gDMRs, we found methylation acquisition specifically in the oocyte, and protection of maternally methylated alleles prior to implantation (Figures 5B, S3B, and S7B). However, paternal alleles of these DMRs undergo de novo methylation at implantation, so that both parental alleles displayed similar levels of methylation at 9.5 dpc as well as in 17.5 dpc fetal liver (Table 1). Contrary to imprinting-associated gDMRs, which show lifelong parental differences at least in some tissues, these DMRs may be categorized as transient gDMRs. Interestingly, in *Dnmt3L-/+* embryos, paternal and maternal alleles were equally methylated at 9.5 dpc, suggesting no differential treatment of the two alleles (Figures 5B and S7B). The global methylation per parental allele was slightly lower than age-matched wild-type embryos, likely as a consequence of a postimplantation developmental delay (Bourc’his et al., 2001). Examination of various normal adult tissues by bisulfite sequencing confirmed complete methylation later in life (Figures 5C and S7C). Further analysis of the last exon of *ZNF777* in human postimplantation tissues confirmed the existence of methylated alleles only (data not shown).

The observation of parent-specific marks at the blastocyst stage prompted us to analyze the allelic methylation of these transient gDMRs in ES cells. Bisulfite analysis showed that ES cells do not reproduce the parental methylation differences of their biological progenitors; the *Zfp777* DMR was biallelically methylated, while the *Zfp787* DMR was biallelically hypomethylated (Figures 5D and S7C). This was observed in ES cells that were isolated and grown in conditions optimal for “ground-state” pluripotency cells (2i medium) (Nichols et al., 2009), and in ES cells cultured in classical medium (data not shown). Similar to previous findings (Borgel et al., 2010; Dean et al., 1998), our results demonstrate that ES cells do not necessarily maintain the allelic status of sequences that are differentially methylated in the blastocyst, and specifically, may not be a suitable cellular model for studying transient gDMRs.

When allelic expression patterns were measured in hybrid reciprocal tissues, we found no parental bias in *Zfp777* and *Zfp787* expression, even in the preimplantation blastocyst, where parental methylation differences still exist (Figures 5E and S7D). Our results suggest that the methylation located in the 3′ end of the *Zfp777* and *Zfp787* genes may not functionally impinge on their expression.

## Discussion

In mammals, the oocyte and sperm genomes harbor distinct methylation patterns, as a result of different kinetics and constraints exerted on gamete production in the two sexes. The inheritance of parent-specific methylation at fertilization provides the opportunity for differential allelic regulation in the progeny, with genomic imprinting as the most durable form of parent-specific regulation of gene expression. Our present work demonstrates that the total number of maternal germline DMRs persisting throughout development and adulthood is very limited, in line with current estimates for the number of known ICRs. From this study, it can be concluded that genomic imprinting is an unusual form of regulation in mammals.

**Figure 6:**
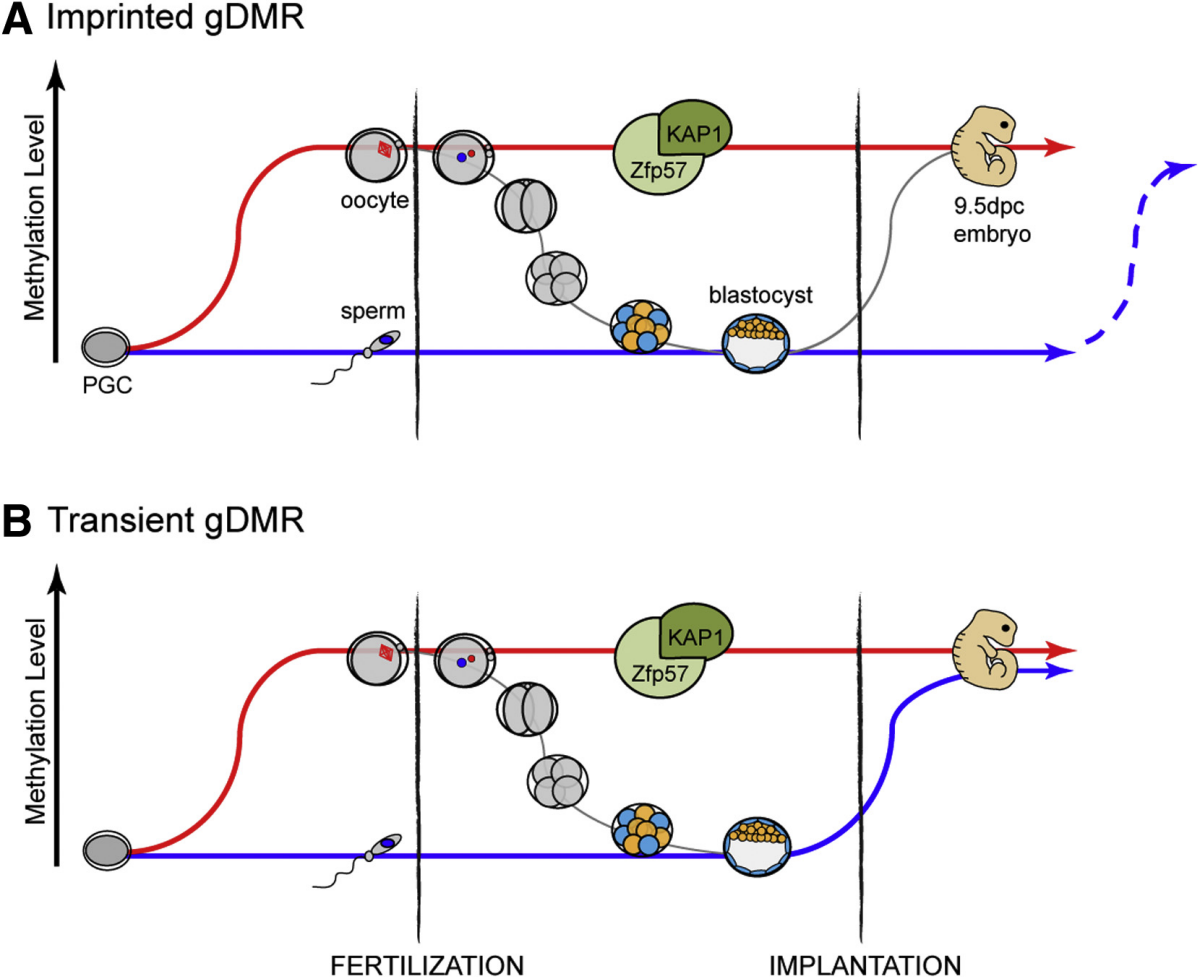
Different Fates of Oocyte-Inherited Methylation (A and B) Maternal alleles (red line) of (A) imprinted and (B) transient gDMRs acquire methylation in oocytes and are protected against genome-wide (gray line) demethylation during preimplantation development. These two types of gDMRs contain Zfp57 binding sites and physically interact with Zfp57/KAP1. However, while unmethylated paternal alleles (blue line) of imprinted gDMRs are protected against de novo methylation at implantation, transient gDMRs are permissive to this process. Imprinted gDMRs can also gain methylation on paternal alleles later during life, in a tissue-specific manner (dotted blue line).

Recent genome-wide studies have highlighted preimplantation demethylation as a major determinant of gametic methylation clearance (Borgel et al., 2010; Kobayashi et al., 2012a; Smallwood et al., 2011). We reveal here that de novo methylation plays an equally important role in leveling parental methylation differences inherited from the gametes (Figure 6). *Zfp777* and *Zfp787* DMRs lose their maternal specificity early, by paternal methylation acquisition at implantation. *Cdh15* and *AK008011* DMRs are protected at implantation, but nevertheless gain paternal methylation later, during tissue differentiation. The permanency and universality of imprinted gDMRs was a commonly held notion in genomic imprinting. The tissue-specificity of *Cdh15* and *AK008011* gDMRs revisits this notion and highlights the limitation of studies performed on a specific adult tissue for identifying new imprinted gDMRs. In this regard, a recent genome-wide screen performed on adult mouse cortex uncovered nine candidate regions of parent-of-origin methylation (Xie et al., 2012), of which only two candidates overlapped with our unfiltered candidate list (*AK008011* and *Casc1*). Interestingly, loss of parent-specific marks by de novo methylation may not be restricted to the new imprinted loci we describe: indeed, at traditionally known ICRs, loss of differential methylation has been sporadically reported in normal adult cells, occurring by methylation gain, rather than loss (Fang et al., 2012; Ferrón et al., 2011).

Our work increases the number of known imprinted gDMRs to 23, including two new loci to be referenced. While the *Cdh15* DMR is associated with parent-specific expression, the *AK008011* DMR may not be functional, showing that imprinted gDMRs may not necessarily be selected for a role in gene regulation. Previous studies had alluded to a possible imprinted status of *Cdh15*. Analysis of chromosome 8 duplications led to the characterization of a region of complete maternal methylation and intermediate paternal methylation in embryos and neonates (Kelsey et al., 1999). While no parent-specific *Cdh15* expression was found in embryos, a recent analysis reported paternal-specific expression of three SNPs confined to exons 12 to 14, in adult hypothalamus (Gregg et al., 2010). Our study resolves the *modus operandi* of this locus, by the identification of a maternal gDMR that maps to *Cdh15* exons 10–12, which is maintained in a tissue-specific manner and controls the paternal expression of a short alternative transcript in neonatal brain and adult hypothalamus. The *Cdh15* DMR may be a docking site for transcription factors expressed in specific brain cell types, whose binding/activity is impaired by maternal DNA methylation.

Cdh15/M-cadherin is an adhesion protein that mediates cell-to-cell interactions. Homozygous *Cdh15* null mice are viable, and show no apparent defects in skeletal muscle and cerebellum, likely due to compensation from other cadherins (Hollnagel et al., 2002). Moreover, there is no evidence of parent-of-origin effects in these mutant mice. However, the corresponding deletion targets exons 1 to 4, and therefore, should not impair the production of the short imprinted *Cdh15* transcript. Interestingly, a similar 5′ truncated form of cadherin with altered adhesion activity has been described in specific neurons of the chick embryo (Shirabe et al., 2005). Provided that the short imprinted *Cdh15* transcript is translated, it may likewise exert specific functions in mammalian hypothalamic cells, related to cell communication, polarization and shaping.

By identifying both imprinted and transient gDMRs, our screen highlights that these two types of gDMRs are indistinguishable in gametes and early embryos (Figure 6). Recruitment of KAP1 through Zfp57 binding was shown to be required for the maintenance of methylated alleles of ICRs (Li et al., 2008; Mackay et al., 2008; Quenneville et al., 2011; Zuo et al., 2012). By in silico analysis we found that all the maternal gDMRs we validated (*Slc38a4*, *Cdh15*, *AK008011*, *Zfp777* and *Zfp787*) contain some hexanucleotide motifs for Zfp57 binding (Table S1). Moreover, Zfp57 and KAP1 are enriched at these sites in published ES cell ChIP-Seq data (Table S1) (Quenneville et al., 2011). Our study therefore shows that the presence of Zfp57 motifs cannot be used as a hallmark of genomic imprinting, as it is also found at transient gDMRs. However, it is likely to specify all genomic sequences that maintain gametic methylation during preimplantation development. Interestingly, four Zfp57 binding motifs exist at the intragenic CGI of the human *CDH15* locus. While we found no evidence of imprinting, we cannot exclude that this locus is a true maternal gDMR in human, either transient or tissue-specific.

Methylation gain at implantation is what discriminates transient from lifelong imprinted gDMRs. The former are permissive, while the latter are refractory to this process. *Zfp777*, *Zfp787*, and *Cdh15* DMRs are all intragenic sequences, for which a strong positive correlation has been reported between DNA methylation and transcriptional read through from the host gene (Ball et al., 2009; Chotalia et al., 2009). Paternal de novo methylation at transient *Zfp777* and *Zfp787* gDMRs may therefore be facilitated by ongoing transcription from these genes at implantation. Conversely, at the imprinted *Cdh15* gDMR, low levels of transcription from the main upstream promoter, local enrichment in H3K4 methylation and transcription factor occupancy may protect from de novo methylation (Lienert et al., 2011; Ooi et al., 2007). The same rules would apply later during life with tissue formation. The DMR is conserved in tissues where it acts as an active promoter for the short paternal *Cdh15* transcript (hypothalamus and neonatal brain), and is potentially protected by transcription factors and/or H3K4me marks. However, in tissues where the short transcript is not expressed, different methylation states are observed and seem to correlate with the expression status of the long canonical *Cdh15* transcript.

Our screen designed at 8.5 dpc was effective at identifying tissue-specific imprinted gDMRs, because it was performed at a time when they are still universal. It also identified transient gDMRs during their remethylation process. Although the transient gDMRs we found do not seem to affect expression, presumably because of their 3′ position, other transient gDMRs may regulate the transcriptome of the peri-implantation embryo. Notably, the parental specificity of these methylated sequences should be lost upon somatic nuclear transfer, resulting in two methylated alleles instead of one during preimplantation development. Furthermore, as for imprinted gDMRs, transient gDMRs may be sensitive to assisted reproductive technologies, involving stimulation of oocyte production and preimplantation embryo culture.

## Experimental Procedures

### MeDIP-Seq

MeDIP-Seq was performed on three pooled litters with the *Dnmt3L+/+* (WT) or *Dnmt3L-/+* genotype. All MeDIP and sequencing library preparations were performed in parallel. Additionally, MeDIP-Seq was performed on three independent C57Bl6/J sperm samples and twelve pools of three livers each of 17.5 dpc fetal hybrid C57Bl6/J and PWD/PhJ mice. MeDIP enrichment and preparation of paired-end sequencing libraries were then performed as described (Down et al., 2008), using monoclonal anti-5-methylcytosine antibody (Eurogentech) and magnetic anti-mouse beads (Dynabeads) for immunoprecipitation. All libraries were sequenced using an Illumina GA2x instrument.

### DNA Methylation Analyses

For MSRE-qPCR, the methylation-dependent restriction enzyme *McrBC* was used, and methylation percentages were calculated according to (Oakes et al., 2009). Values represent the average of three independent digestion experiments, performed on DNA from 8.5 dpc litters of eight embryos. For bisulfite conversion, DNA was treated with the EpiTect kit (QIAGEN). BiQ Analyzer software was used for sequence alignments (Bock et al., 2005) and clones with identical patterns of conversion were removed from the final pileup.

## Figures and Tables

**Table 1:** Maternal gDMR Candidates: Hypomethylated, Single-Copy, and Relatively CpG-Rich DMRs

Chr	Start	End	Closest Transcript	CGI	CpG Content	Sperm Methylation^a^	Liver Methylation^b^
8	125387861	125390344	Cdh15	CGI21235	0.37	−5.1	0.1334
6	47974007	47975979	Zfp777	CGI17361	0.56	−5.3	0.6911
13	54209856	54211139	Sfxn1		0.25	−0.9	*0.5000*
17	87524084	87525819	Socs5	CGI9617	0.43	−3.4	0.0020
7	86519538	86521097	Rlbp1		0.23	−0.9	0.1334
7	6083480	6084890	Zfp787	CGI18282	0.64	−4.7	0.6964
7	24992450	24993377	Zfp111	CGI18528	0.45	−2.7	*0.7500*
15	76010966	76012080	Plec1	CGI7215	0.68	−1.5	*1.0000*
11	102057005	102057998	Hdac5		0.30	−1.2	*0.0078*
13	60557950	60559042			0.24	−1.1	*1.0000*
16	20530221	20531293	Dvl3	CGI8073	0.27	−1.9	*0.3438*
4	150993001	150994022	Camta1		0.33	−2.6	*1.0000*
4	53727006	53728024	Fcmd	CGI14304	0.89	−3.5	*0.8750*
15	102047274	102048271	Itgb7		0.26	−1.3	*0.7500*
5	106629403	106630408	nenese		0.66	−4.7	*0.3125*
10	122303419	122304463	Ppm1h		0.26	−1.4	0.0384
7	148034494	148035458	Odf3		0.26	−1.0	*1.0000*
7	147267611	147268611	Drd1ip	CGI19894	0.34	−2.2	*1.0000*
15	11250512	11251415	Adamts12		0.29	−1.4	*1.0000*
13	66815007	66816022	2410141K09Rik		0.57	−2.9	*1.0000*
14	122056331	122057326	Dock9		0.32	−0.8	*1.0000*
8	12262778	12263827			0.21	−1.1	*1.0000*
12	118489501	118490705	Ptprn2		0.30	1.3	0.5000
19	45385459	45386455	sneefar		0.27	−0.8	*0.7734*
6	125660898	125661895	Tmem16b	CGI18014	0.28	−1.0	1.0000
11	115748842	115749776	Myo15b		0.22	−1.2	0.5000
10	74869979	74870980	Upb1		0.28	−2.2	1.0000
13	96588183	96589194	Iqgap2		0.27	−0.8	0.1938

Information shown: genomic coordinates of the DMR as determined by USeq, closest transcript (RefSeq), CGI reference number (Illingworth et al., 2010), observed/expected CpG ratio, methylation status in sperm, and evidence for maternal methylation in hybrid fetal liver.

a log_2_ of fold-change relative to wildtype embryos: negative values are indicative of sperm hypomethylation.

b one-tailed binomial test p values: entries with p > 0.2 are indicative of low degree of evidence for maternal-specific methylation; italics highlight entries where the test was underpowered.

## Accession Numbers

Raw MeDIP-Seq read and alignment data are available from GEO with accession GSE32687.

## Supplemental Information

Document S1. Seven Figures, Two Tables, Supplemental Experimental Procedures, and Supplemental References

Table S1. DMRs Identified by MeDIP-Seq between *Dnmt3L-/+* and Wild-Type 8.5 dpc Embryos
