# Supplementary material for: Protection against De Novo Methylation Is Instrumental in Maintaining Parent-of-Origin Methylation Inherited from the Gametes
Source: Mol Cell. 2012 Sep 28;47(6):909–20. doi: 10.1016/j.molcel.2012.07.010 (PMC3778900; doi:10.1016/j.molcel.2012.07.010)
Supplement: Document S1. Seven Figures, Two Tables, Supplemental Experimental Procedures, and Supplemental References [file mmc1.pdf]

Molecular Cell, Volume *47*

Supplemental Information

Protection against De Novo Methylation Is Instrumental in Maintaining Parent-of-Origin Methylation Inherited from the Gametes

Charlotte Proudhon, Rachel Duffié, Sophie Ajjan, Michael Cowley, Julian Iranzo, Guillermo Carbajosa, Heba Saadeh, Michelle L. Holland, Rebecca J. Oakey, Vardhman K. Rakyan, Reiner Schulz, and Déborah Bourc'his

Supplemental Information Inventory

**Supplemental Figure/Table**	**Related main Figure/Table**	**Information**
Figure S1	Figures 1A, 1B, 1C, 1D	MeDIP-Seq profiles of known imprinted loci controlled by maternal ICRs, as positive controls of the sensitivity of the screen
Figure S2	Figures 1A 1D, and 1E	MeDIP-Seq profiles of known paternal ICRs and germline genes, as controls of the specificity of the screen
Figure S3	Figures 1A, 1E, 2B and 5B	Germline methylation profiles of candidate maternal gDMRs
Figure S4	Table 1	Investigation of allelic methylation in 17.5dpc fetal liver
Figure S5	Figure 2, 3E, 3F	Further characterization of *Cdh15* imprinting in mouse and human
Figure S6	Figure 3	Lack of imprinted expression of genes in the vicinity of *Cdh15*
Figure S7	Figure 5 and Table 1	Identification and methylation analysis of a second transient maternal gDMR, associated with *Zfp787*
Table S1	Figures 1, 2, 4, 5, 6, and Table 1	DMRs identified by MeDIP-Seq between *Dnmt3L-/+* and wildtype 8.5dpc embryos
Table S2	Figures 1, 2, 3, 4, 5	Primer list
Table S3	Figures 3B, 3C	Antisera

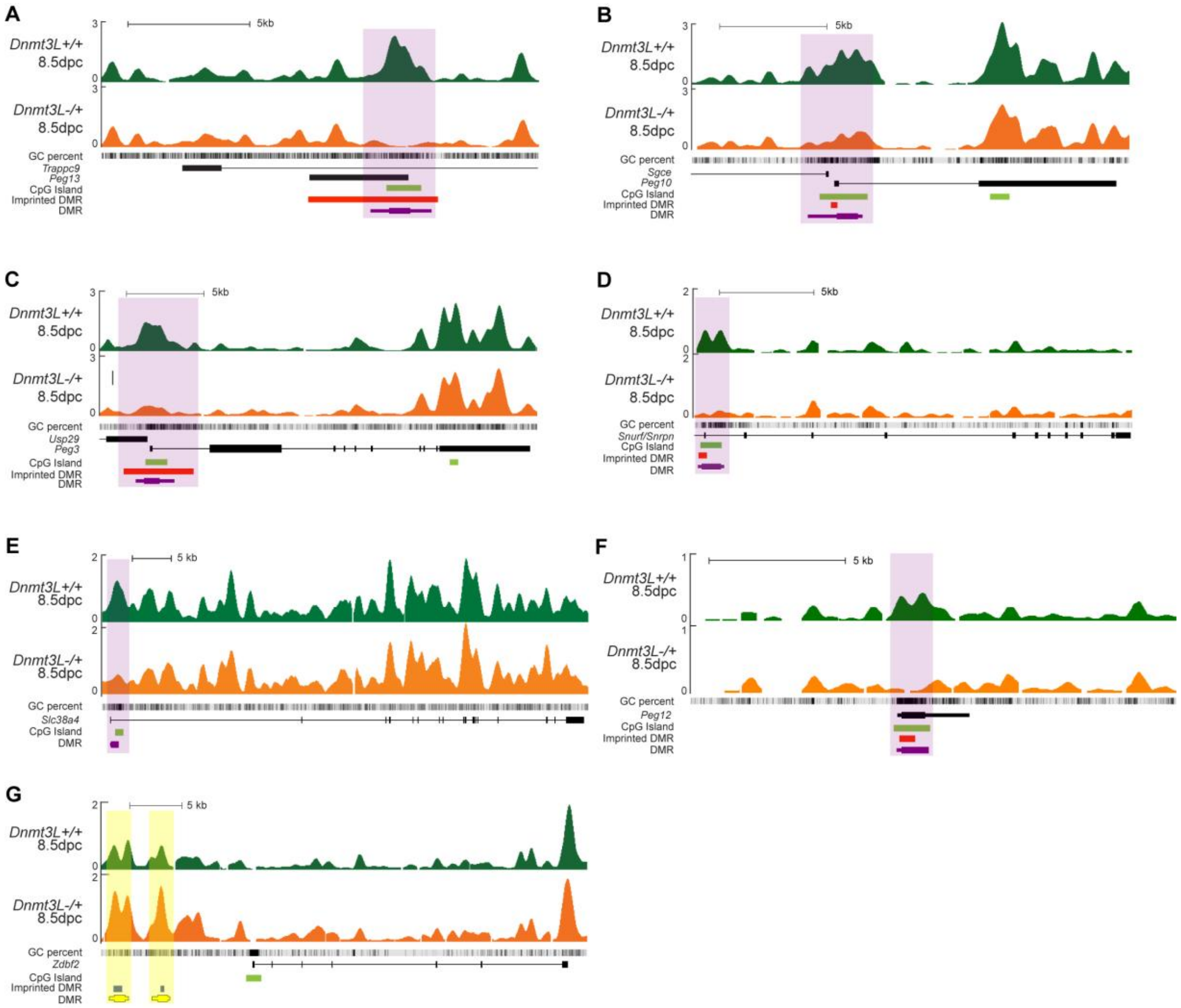

Proudhon, Duffié_SupFigS1

Figure S1. MeDIP-Seq profiles of known imprinted loci controlled by maternal ICRs, as positive controls of the sensitivity of the screen

Known maternal ICRs associated with *(A) Peg13*, the highest ranking locus of the screen, and with *(B) Peg10/Sgce*, the lowest ranking ICR of the screen. *(C) Peg3* ICR, and *(D) Snrpn/Snurf* ICR, two loci that were reported to sporadically regain methylation in *Dnmt3L-/+* embryos, also show significant hypomethylation by MeDIP-Seq. The screen allowed the improved mapping of regions of Dnmt3L-dependent methylation in partially documented imprinted loci, such as (*E) Slc38a4* and (*F) Peg12*. (*G)* Two hypermethylated DMRs (yellow), located upstream of the *Zdbf2* gene, coincide with two regions previously reported to be paternally imprinted gDMRs (blue). Their gain of methylation in *Dnmt3L-/+* embryos instead indicates that they are secondary somatic DMRs, under the control of a primary maternal gDMR.

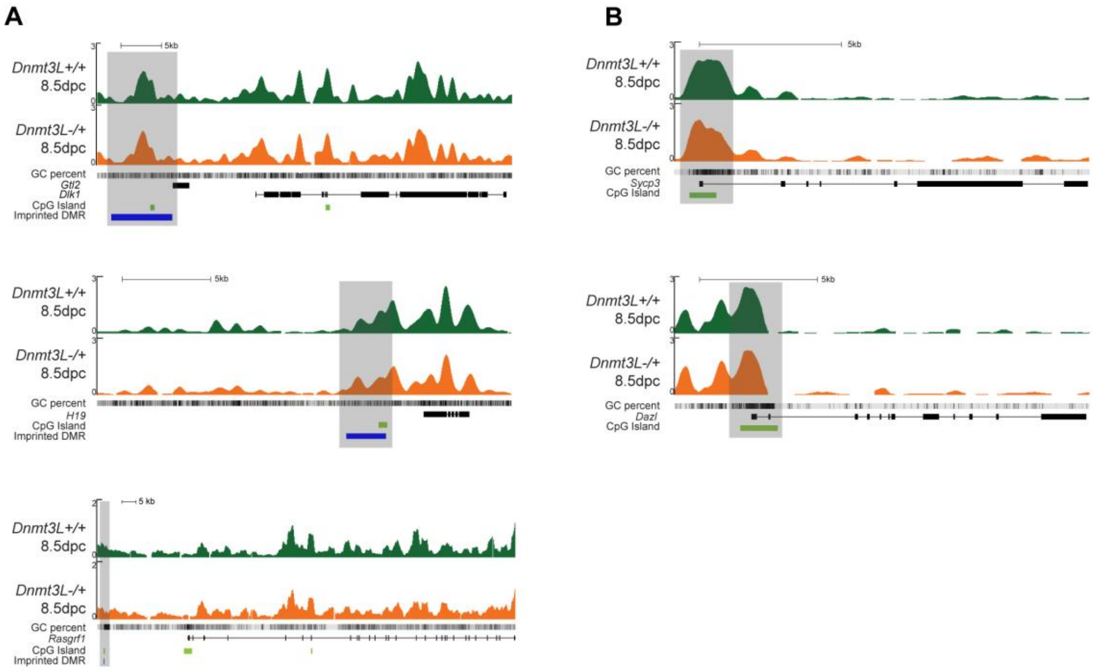

Proudhon,Duffié_SupFigS2

Figure S2. MeDIP-Seq profiles of known paternal ICRs and germline genes, as controls of the specificity of the screen

Methylation at these loci is not altered in *Dnmt3L-/+* embryos (gray). *(A)* Known paternal ICRs (blue), associated with *Dlk1-Gtl2* (top), *H19-Igf2* (middle), and *Rasgrf1* (bottom) loci. These gDMRs depend on sperm-inherited DNA methylation. *(B)* Promoters of germline-expressed genes, associated with the *Sycp3* gene (top) and the *Dazl* gene (bottom). These regions acquire methylation somatically, in the embryo at the time of implantation, and do not depend on oocyte-inherited methylation.

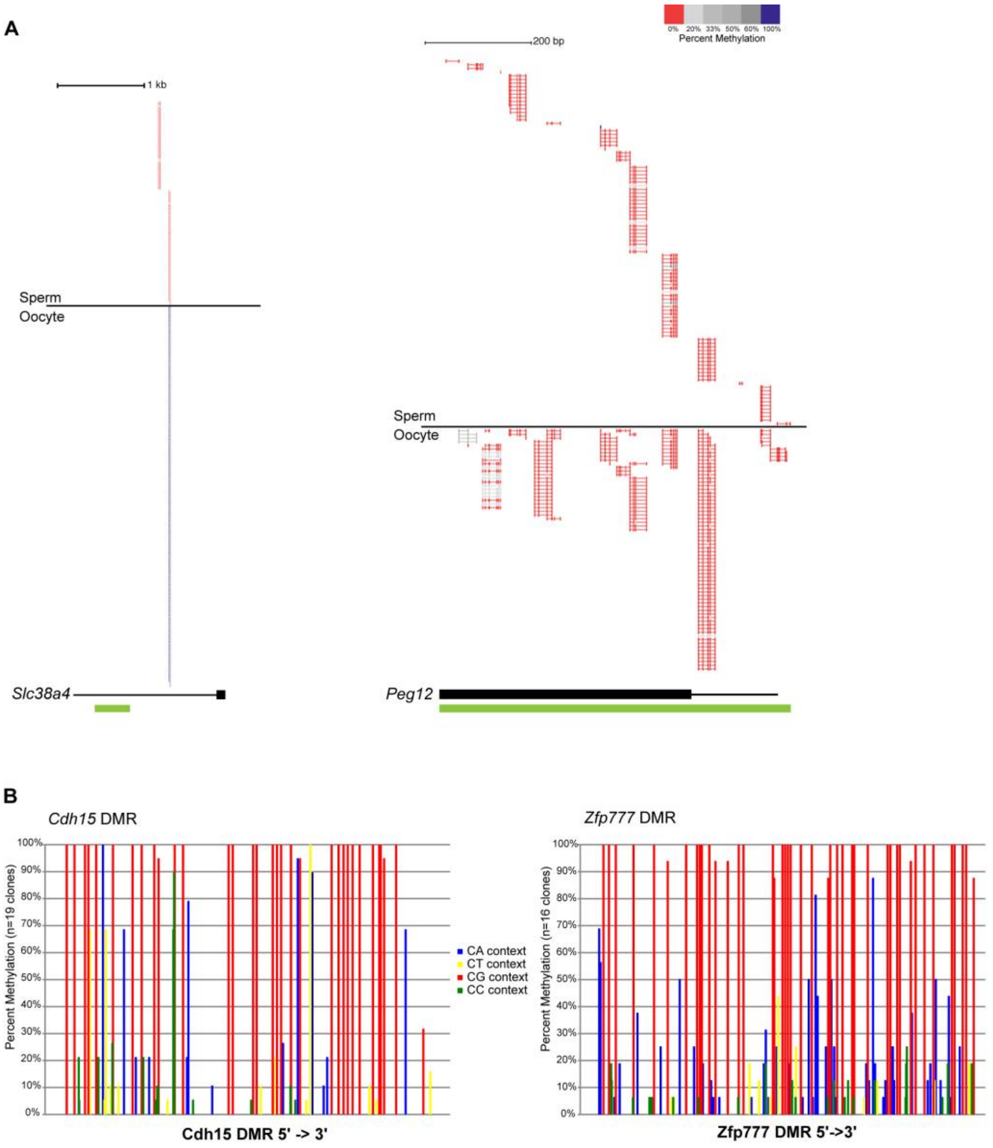

Proudhon, Duffié_SupFigS3

Figure S3. Germline methylation profiles of candidate maternal gDMRs
RRBS data showing that *(A)* the *Slc38a4* DMR is specifically methylated in oocytes but not in sperm, and therefore classifies as a maternal gDMR, while *(B)* the *Peg12* DMR is unmethylated both in oocytes and sperm and is therefore not a gDMR. Blue=methylated, red=unmethylated, green=CpG island. *(C)* Detection of oocyte non-CG methylation by bisulfite sequencing at the imprinted maternal gDMR of *Cdh15* and at the transient maternal gDMR of *Zfp777*.

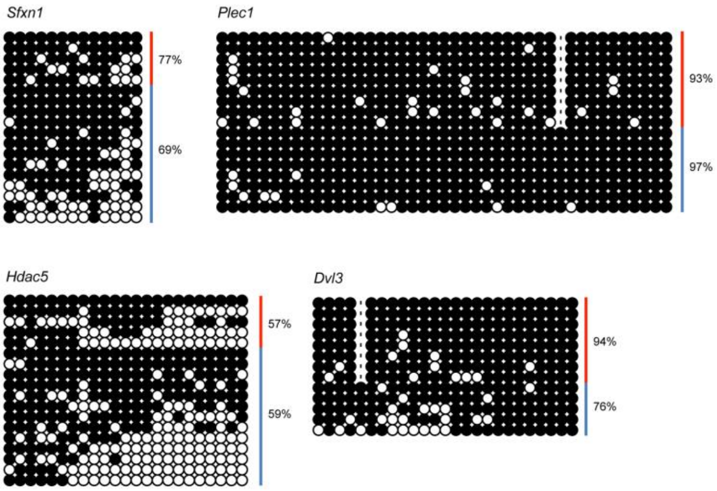

Proudhon, Duffié_SupFigS4

Figure S4. Investigation of allelic methylation in 17.5dpc fetal liver
DNA from BxC crosses was used for bisulfite sequencing of four maternal gDMR candidates (*Sfxn1*, *Plec1*, *Hdac5* and *Dvl3*), which failed to reach statistical significance because of low read count in fetal liver MeDIP-Seq data. All of them showed equal methylation on maternal and paternal alleles, demonstrating that maternal-specific methylation is not maintained at these loci in this post-implantation tissue.

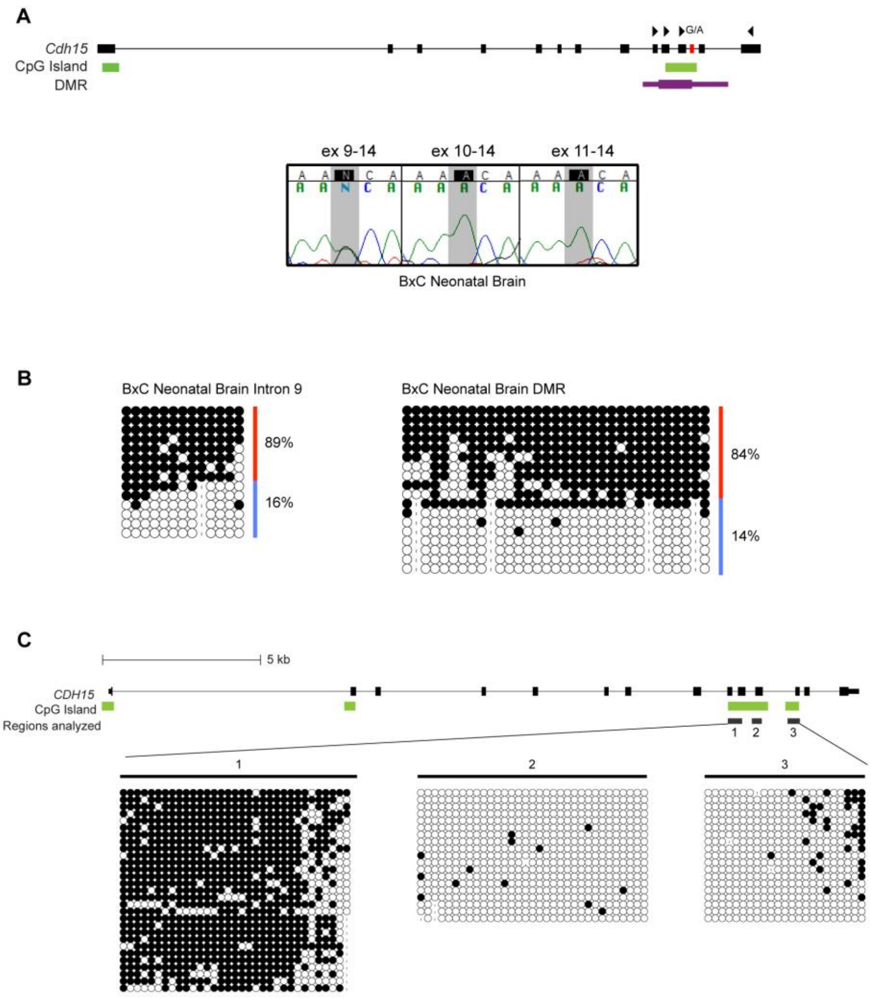

Proudhon, Duffié_SupFigS5

Figure S5. Further characterization of *Cdh15* imprinting in mouse and human
(A) Mapping of the short *Cdh15* transcript in mouse by RT-PCR sequencing reveals a switch from biallelic to monoallelic expression between exons 9 and 10, placing the origin of the short *Cdh15* imprinted transcript around intron 9. Amplifications were performed on BxC and CxB neonatal brain RNA, where both the canonical and the short *Cdh15* transcript isoforms are expressed, using a reverse primer in exon 14 and forward primers in exons 9, 10 or 11. The A/G SNP is located in exon 12 (red). Arrow heads indicate primer positions. *(B)* In mouse neonatal brains, maternal-specific methylation is observed both at the putative TSS of the short imprinted *Cdh15* transcript (intron 9) and at the DMR. *(C)* In humans, the region corresponding to the mouse *Cdh15* DMR also overlaps with a large CGI. We analyzed three parts of this CGI in fetal liver DNA. Region 1 was found globally methylated, with no obvious allelic specificity, as seen with an informative SNP. The most 3' part of Region 1 showed a decrease in methylation. Regions 2 and 3 located downstream were found completely unmethylated, again with no evidence of allelic specificity, although no SNP was found in these amplified regions.

Acsf3 (exon 2)

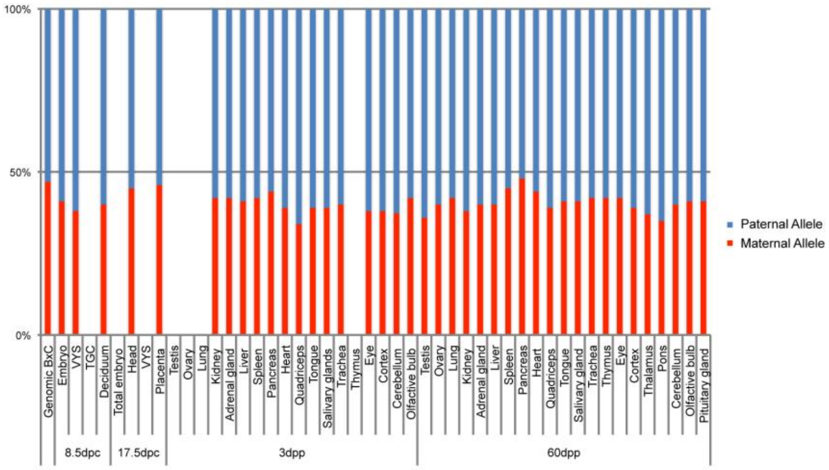

Ak040202 (3'UTR)

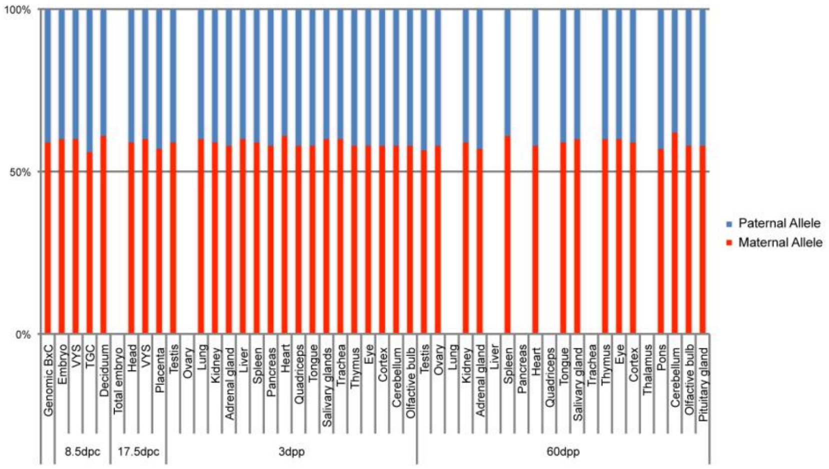

Ankrd11 (exon 10)

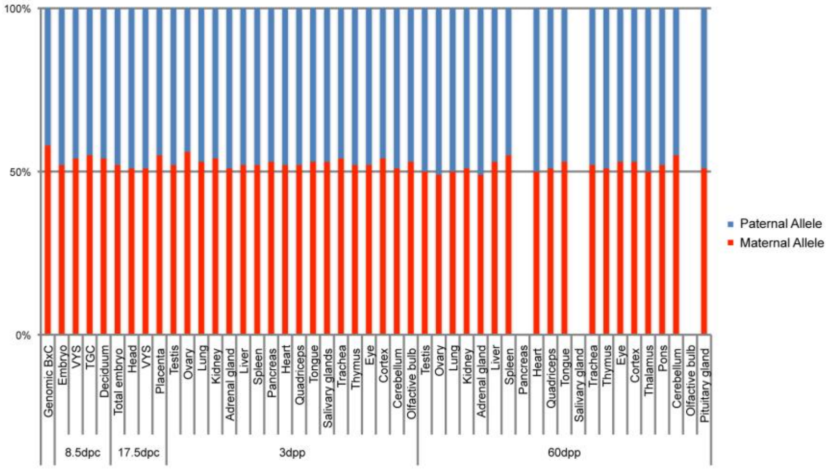

Proudhon, Duffié_SupFigS6

Figure S6. Biallelic expression of *Asf3*, *Ankrd11* and *AK040202*, located in the vicinity of *Cdh15*

RT-PCR pyrosequencing was performed on a bank of 47 tissues from reciprocal BxC and CxB crosses. Results are presented for one orientation of the cross only. Locations of SNPs are indicated after each gene name. Missing bars indicate no detected expression.

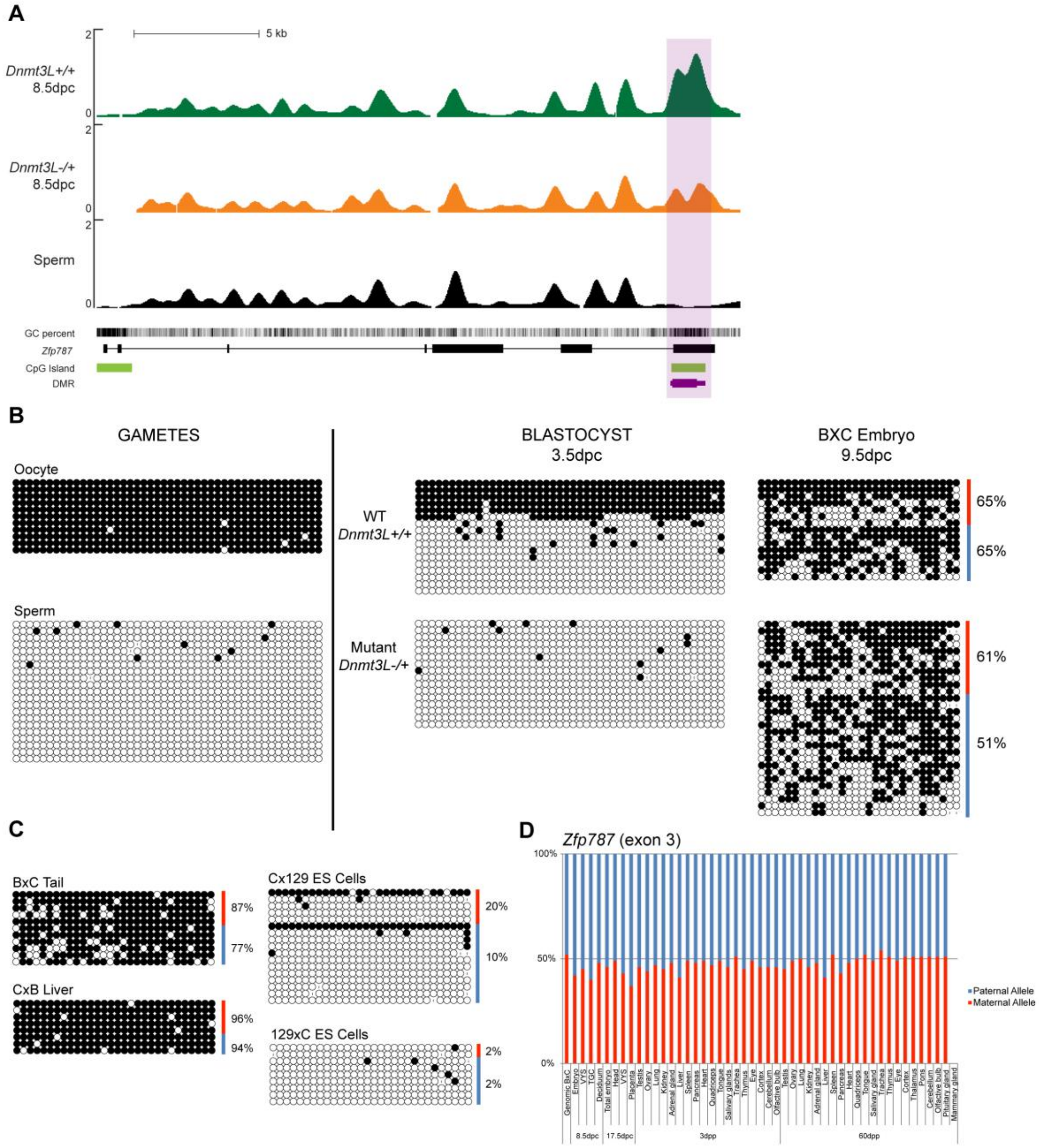

Proudhon, Duffié_SupFigS7

Figure S7. Identification and methylation analysis of the transient maternal gDMR associated with *Zfp787*

(A) MeDIP-Seq profile of the *Zfp787* locus. Genes are oriented 5' to 3'. The hypomethylated DMR maps to a CGI in the last exon of the *Zfp787* gene. *(B)* Developmental analysis of *Zfp787* DMR methylation by bisulfite sequencing. This region acquires its methylation in oocytes but not in spem, maintains differential methylation during preimplantation, but is completely unmethylated in *Dnmt3L-/+* blastocysts. Contrary to imprinted gDMRs, methylation is gained on paternal alleles at implantation and parental alleles exhibit similar methylation levels at 9.5dpc, both in WT and *Dnmt3L-/+* embryos. *(C)* Adult hybrid tissues (tail and liver) show a fully methylated pattern. In opposition to the blastocyst, both parental alleles are unmethylated in ES cells. *(D)* A RT-PCR pyrosequencing assay was designed to study a SNP located in *Zfp787* exon 3. The *Zfp787* gene is biallelically expressed throughout development and adulthood.

Table S1. DMRs identified by MeDIP-Seq between *Dnmt3L-/+* and wildtype 8.5dpc embryos

DMRs were identified using a sliding window approach (See Material and Methods). We carried out the analysis twice, using two different window sizes: 1000bp and 500bp. Each window size is reported on a separate sheet (named “1000bp” and “500bp”). *Columns A-F*: percent of the DMR covered by a particular repeat class (Satellite, Sine, Line, etc.). *Columns G & H*: total percent retrotransposon and repetitive sequence content, respectively. Red highlights indicate high repeat content (>25% and >50%). *Columns I-L & K*: overlaps of the DMR with (AceView) transcripts, split into promoters, 3'UTR, exons and introns, and overlap with known (red, blue) or validated (purple, yellow) gDMRs/ICRs. *Columns N-T*: DMR $\log_2$ fold-change in methylation (cyan: hypomethylation; magenta: hypermethylation), FDR, genomic coordinates (Q & T: start and end of entire region meeting FDR<50% threshold; R & S: start and end of 1Kbp/500bp sub-window where the most differential methylation was observed (thick part of bar representing DMRs in Figures) and on which the FDR calculation is based). *Columns U-X*: counts of CGs, Cs and Gs within DMR, and the corresponding observed over expected #CG ratio. *Columns Y-AE*: overlaps of DMR with ChIP-seq peaks for Zfp57+Kap1+Setdb1 reported in (Quenneville et al., 2011), midpoint distance between DMR and peak, peak genomic coordinates, number of occurrences of Zfp57 binding motif within DMR. *Columns AF-AM*: genomic position of SNP between C57Bl6/J and PWD/PhJ used for binomial test, distance of SNP to DMR midpoint, number of reads overlapping SNP, number of reads from reference allele (C57Bl6/J), number of reads from alternative allele (PWD/PhJ), one-sided binomial test p-value for H1: better than 50% chance of maternal methylation, one-sided binomial test p-value for H1: better than 50% chance of paternal methylation, position and allele counts for all SNPs within DMR. *Columns AN-AQ*: $\log_2$ fold-change in methylation for DMRs between sperm and wildtype 8.5dpc embryos that most overlap with DMRs between *Dnmt3L-/+* and wildtype embryos, FDR for sperm vs wildtype DMRs, percent of *Dnmt3L-/+* vs wildtype DMR covered by sperm vs wildtype DMRs, $\log_2$ fold-change and FDR for all sperm vs wildtype DMRs that overlap the *Dnmt3L-/+* vs wildtype DMRs. *Columns AR-AT*: distance to closest exon showing allelic expression bias in data from (Gregg et al., 2010), parental allele with higher expression, binomial test confidence score ($-10\log_{10}$ p-value). *Columns AU-BA*: distance to closest promoter investigated for histone modifications by (Mikkelsen et al., 2007), gene name, RefSeq accession, promoter class (H/I/LCP = high/intermediate/low CpG content), observed modifications in ESCs, NPCs and MEFs. *Columns BB-BO*: overlapping peaks of histone modifications called by (Mikkelsen et al., 2007) using two different methods (BB-BF: HMM method; BG-BO: sliding window method). *Columns BP-BR*: distance to closest (Hiura et al., 2010) DMR candidate by MeDIP-chip (promoter array), p-value and fold-change as reported. *Columns BS-CN*: distance to closest CGI reported in (Illingworth et al., 2010), followed by annotation from the original publication. *Columns CO-CW*: Average percent methylation of overlapping CGIs, identified by CGI ID from (Illingworth et al., 2010) and/or genomic coordinates, with sufficient BS-seq data in wildtype oocytes, *Dnmt3L-/-* oocytes, sperm, blastocysts and mouse ES samples, as reported in Kobayashi et al., 2012a.

Table S2. Primer list

**Bisulfite**		
Sfxn1-outer	Fwd	5'- TTTGGGAGTTTTGAGTATTTGAG -3'
	Rev	5'- ATAACTCCTCTCTAAAAATAAATTC -3'
Sfxn1-inner	Fwd	5'- TTTGGGAGTTTTGAGTATTTGAG -3'
	Rev	5'- CTCATCTAAAATTATTATTCAAAATC -3'
Plec1-outer	Fwd	5'- TTTTGTTGGTTAGAAGTATTTTTAT -3'
	Rev	5'- ACCCTAAATAAACTAAAACTACAA -3'
Plec1-inner	Fwd	5'- GATTTTAGTTAGTTTAGTTTTTAG -3'
	Rev	5'- CCAAATACAAATAACCCTAAAAA -3'
Dvl3-outer	Fwd	5'- GGATTAGTTGTAGAGTTATTGAG -3'
	Rev	5'- ACTCCTAAAAAACCCATAACTAC -3'
Dvl3-inner	Fwd	5'- GTAGTTTATTTTTGGTTGGAGATT -3'
	Rev	5'- CCATAACTTAAATATATATAATAACTA -3'
Hdac5-outer	Fwd	5'- AGATAGAGAGGAAGGGAAAGT-3'
	Rev	5'- TCCTCATCCCACTCTAAACCT-3'
Hdac5-inner	Fwd	5'- GAGGGTGGAGAGGGGTAGAT-3'
	Rev	5'- TTCTCTACCAACTACAACCCTTA-3'
Cdh15-outer	Fwd	5'- TTATGTTTTTGTATTGGTTTTTTTTT -3'
	Rev	5'- AAAATAAAAAAAAAACCCAAAACAAA
Cdh15-inner	Fwd	5'- TTAGTTGAATTTTAGAGTATTAGATT -3'
	Rev	5'- AAATAAAACCAAAAACCTTCACTCAC -3'
Cdh15 intron9-outer	Fwd	5'- TAGAAGATTGGTTGTAAGTGGA-3'
	Rev	5'- ACCTATCTCTTAAATCTCAAAATTA-3'
Cdh15 intron9-inner	Fwd	5'- GTTGGTATAGAGTTATTATTTTAG-3'
	Rev	5'- ACCTATCTCTTAAATCTCAAAATTA-3'
AK008011-gSNP-outer	Fwd	5'- GTAGAGGGAGTATTAGGAAGG -3'
	Rev	5'- CACATCATCAAACTCTATAACAAA -3'
AK008011-gSNP-inner	Fwd	5'- GTTATTATAGTATTGATTATTTTTTA -3'
	Rev	5'- AGAATTTAATTATTGGGTTTTTTG -3'
Zfp777-Bl6-outer	Fwd	5'- TAAATGTTTTGAGTGTGATAGTAG -3'
	Rev	5'- CCTTACACCTAACTACACATCTAA -3'
Zfp777-Bl6-inner	Fwd	5'- AGTTAAGTTTGATTAAGTATTAGATT -3'
	Rev	5'- CCTTACACCTAACTACACATCTAA -3'
Zfp777-gSNP-outer	Fwd	5'- ATAGATTGTAAATGAAGGAGA -3'
	Rev	5'- CACCCAACATTCATAAACATA -3'
Zfp777-gSNP-inner	Fwd	5'- GTGGATTATTAGATTGATATG -3'
	Rev	5'- CACCCAACATTCATAAACATA -3'
Zfp787-Bl6-outer	Fwd	5'- AGATGTGTGTAGGTTTTTTATTAA -3'
	Rev	5'- AAAACCATATACCTACTCAAAATA -3'
Zfp787-Bl6-inner	Fwd	5'- AGATGTGTGTAGGTTTTTTATTAA -3'
	Rev	5'- AAAACTCCAACCTCATACAACACC -3'
Zfp787-gSNP-outer	Fwd	5'- TTGGTGGGTTAATAGTTTAGTT -3'
	Rev	5'- ATATAACAAAAACTTCAACCACT -3'
Zfp787-gSNP-inner	Fwd	5'- ATTTGGTTTTGATAGTTTTGGG -3'
	Rev	5'- ATATAACAAAAACTTCAACCACT -3'
hCDH15-R1-outer	Fwd	5'- TTTTGGGGTAAATTTAGATTTTATT -3'
	Rev	5'- TAACCTAACTAAAACTCCAATTC -3'

hCDH15-R1-inner	Fwd	5’- TTTTGTATTAGTTATTTTAAGGATTA -3’
	Rev	5’- AGTTATATTAAGGTTTAGGTTTTTT -3’
hCDH15-R2-outer	Fwd	5’- TGGGGTAGGGTTATTTATTG -3’
	Rev	5’- TCACTCACCCAACAACAAAA -3’
hCDH15-R2-inner	Fwd	5’- TGGGGTAGGGTTATTTATTG -3’
	Rev	5’- CCCAAACTAAAACCTATACC -3’
hCDH15-R3-outer	Fwd	5’- TTTATTAGTTAAGTTTTTTTTTTTATAA -3’
	Rev	5’- AGGAGTTTGTATTTGAGATTTTTA -3’
hCDH15-R3-inner	Fwd	5’- TTTATTAGTTAAGTTTTTTTTTTTATAA -3’
	Rev	5’- GGAGGAGGATTAGGTGAGG -3’
hZNF777-outer	Fwd	5’- GTGATTAAGATAGAGGAATAAG -3’
	Rev	5’- GTATTATTTGTTGGAGTATTAG -3’
hZNF777-inner	Fwd	5’- ATATGAGGTTAGTATGTATTAG -3’
	Rev	5’- TTTAAGTTATTTAGTTTTGGTAG -3’
**ChIP-qPCR**		
Cdh15DMR-ChIP	Fwd	5’- GAGAGCAAACGCTGAACGTC -3’
	Rev	5’- GGACATTCTGGGACCCCTTT -3’
KvDMR-ChIP	Fwd	5’- AACTCCGAATAAGCAGCCTTC -3’
	Rev	5’- TGGAGTACGTGTTGTTATGTGG -3’
Tbx15-ChIP	Fwd	5’- TCCCCCTTCTCTTGTGTCAG -3’
	Rev	5’- CGGAAGCAAGTCTCAGATCC -3’
IAP-ChIP	Fwd	5’- CTCCATGTGCTCTGCCTTCC -3’
	Rev	5’- CCCCGTCCCTTTTTTAGGAGA -3’
**MSRE-qPCR**		
KvDMR-MSRE	Fwd	5’- AACTCCGAATAAGCAGCCTTC -3’
	Rev	5’- TGGAGTACGTGTTGTTATGTGG -3’
H19-MSRE	Fwd	5’- ACATTCACACGAGCATCCAGG -3’
	Rev	5’- GCTCTTTAGGTTTGGCGCAAT -3’
Nesp-MSRE	Fwd	5’- AGCGCAAGGAGGAAAACAG -3’
	Rev	5’- ATTAGTGACGCCGGATGG -3’
Slc384a-MSRE	Fwd	5’- TCCCTCTCCTCTGAAGTCCTC -3’
	Rev	5’- GAGACTGCTTCCCACATGGT -3’
Peg12-MSRE	Fwd	5’- TGTGCCCTCCATCACAATC -3’
	Rev	5’- CAAAGCTTCCCGCTCACTC -3’
Zdbf2_DMR1-MSRE	Fwd	5’- AACTTTAGAGCCCCAGAAGG -3’
	Rev	5’- CCATCCGACAATTCAAAATC -3’
Zdbf2_DMR2-MSRE	Fwd	5’- ACCCTGGTCCGACCTTATGT -3’
	Rev	5’- GGGAACTCCCATCCTTTAGC -3’
Cdh15DMR-MSRE	Fwd	5’- GAGAGCAAACGCTGAACGTC -3’
	Rev	5’- CCAGCACAATGACCAGTGC -3’
AK008011-MSRE	Fwd	5’- ATGCCGGAACTTGCTCTG -3’
	Rev	5’- GGATGGATGGAGGCTTCTC -3’
Zfp777-MSRE	Fwd	5’- TGAAGCTCTTCTCGCACTCC -3’
	Rev	5’- CCTTCATTTGCAGTCTGTGC -3’
Zfp787-MSRE	Fwd	5’- GGACACACGAAGGGCTTTA -3’
	Rev	5’- GCAGCTTCACACAGAGCAAG -3’
**Northern**		
Cdh15Probe 5-6	Fwd	5’- CTTCGTCACCAGGGCTGAG -3’
	Rev	5’- GGGCATTGTCGTTGATGTC -3’
Cdh15Probe 8-9	Fwd	5’- GGACTATGAGAGCCGTGAGC -3’
	Rev	5’- CTCTGTACCAGCCGTCCTTC -3’

Cdh15Probe 9-14	Fwd	5’- AAGACTGGCTGCAAGTGGAC -3’
	Rev	5’- CTCCCAGGCTGGACAGAAT -3’
**Pyrosequencing**		
Cdh15ex11 (ChIP)	Fwd	5’- (biot)CGAGAGCAAACGCTGAAC -3’
	Rev	5’- CCCAAGCTGACGCCTACAC -3’
	Seq	5’- CAGGCAAGTGCCATC -3’
KvDMR	Fwd	5’- AACGGAGCCCCTCACTCT -3’
	Rev	5’- (biot)GACCCCTGAGCTTTGTAGCC -3’
	Seq	5’- CATTAAAACAGCTACCACAT -3’
Nhlrc1-ex1	Fwd	5’- GGCGTGGTAGCAGGTGAGA -3’
	Rev	5’- (biot)CCTGCCGAGCCTGTGACA -3’
	Seq	5’- AGCCCAGGAGCTCCA -3’
Nhlrc1-3’UTR	Fwd	5’- (biot)ATGTGCTGGGGCAAGATCTA-3’
	Rev	5’- CTGCCTGCTAGCGTCATGG -3’
	Seq	5’- CGTCATGGGCATATGAG -3’
Acsf3-ex2	Fwd	5’- ACTTGGCACTGCCCTTCA -3’
	Rev	5’- (biot)TGGTAGGCAGGAGGCTATGT- 3’
	Seq	5’- TGATTCCCAGAAGACAC -3’
AK040202-3’UTR	Fwd	5’- GGAATGGGCTGGCTTCTGAA -3’
	Rev	5’- (biot)GTGGTCACCTAGGCTGCTTTCTTA -3’
	Seq	5’- GGCTTCTGAAAACAGTG -3’
Ankrd11-ex10	Fwd	5’- (biot)TCTCGAGGTCTTTCTGGGACAG -3’
	Rev	5’- ATAAAGATGAGCAACGGGAACG -3’
	Seq	5’- GGCAAGAAAGACAGCAG -3’
Zfp777-3’UTR	Fwd	5’- GGTCTAAGGCCCCTGGGACA -3’
	Rev	5’- (biot)ACGGGAGGGAAGGAGGGTT -3’
	Seq	5’- CCAGACTGAGCTCAGTG -3’
Zfp787-ex3	Fwd	5’- CACTCTGAGCAGGCATATGG -3’
	Rev	5’- (biot)TACCGACTGCGGCAAGAC -3’
	Seq	5’- GCCGGTGCTGCACCA -3’
Zfp787-3’UTR	Fwd	5’- (biot)GGGGGGCAGAAAAAATAATG -3’
	Rev	5’- TGCATCAAAAGGGCTTCC -3’
	Seq	5’- GAATTTTCTTTTAATCATAC -3’
**RT-PCR**		
Cdh15-ex3SNP	Fwd	5’- GTGTCTGAGAACCACAAACGC -3’
	Rev	5’- CCTCTGAACACATCCTGTAGG -3’
Cdh15-ex14SNP	Fwd	5’ CGTGCCTCCCTACGACACAG -3’
	Rev	5’- GGGCGACTAAGGGTGGCTTC -3’
Cdh15-ex8	Fwd	5’- TCCAGTGTTTCCAGAGAACCC -3’
Cdh15-ex9	Fwd	5’- AAGACTGGCTGCAAGTGGAC -3’
Cdh15-ex10	Fwd	5’- GCTGAACCCCAGAGTACCAG -3’
Cdh15-ex11	Fwd	5’- AGCCACCCCAGCAGCGAGAG -3’
Cdh15-ex13	Rev	5’- ACTGATGAAGTTGGCAATGT -3’
Cdh15-ex14	Rev	5’- CTCCCAGGCTGGACAGAAT -3’
**RT-qPCR**		
Cdh15-ex1qPCR	Fwd	5’- GTCACTCAGCCCAATGCTCT -3’
	Rev	5’- GAGCAGAGCAGAACCCATTG -3’
Cdh15-ex12-13qPCR	Fwd	5’- TGTTGCATGGCCTACAAGAG -3’
	Rev	5’- CGCAGCTGGTTTATGTCGTA -3’
Arp0	Fwd	5’- TCCAGAGGCACCATTGAAATT -3’
	Rev	5’- TCGCTGGCTCCCACCTT -3’

Table S3. Antisera List

	Furnisher	Reference
H3	Abcam	ab1791
H3K4me2	Abcam	ab32356
H3K4me3	Active Motif	39159
H3K9me2	Abcam	ab1220
H3K9me3	Millipore	07-442
H4K20me3	Active Motif	39180

Supplemental Experimental Procedures

Isolation of gametes, embryos and tissues

Dnmt3L+/+ and *Dnmt3L-/+* embryos were obtained at 8.5dpc from a C57Bl6/J background by natural mating, respectively from *Dnmt3L+/+* and *Dnmt3L-/-* females crossed with wildtype males (Bourc'his et al., 2001). Blastocysts were obtained after superovulation and natural mating. Hybrid embryos at 9.5dpc were obtained by crossing Bl6 females with CAST/E1 males. Sperm was collected from the epididymus of 5 week-old C57Bl6/J males. Wildtype oocytes were collected at the MII stage from superovulated B6CBAF1/J females. For allelic expression studies, 47 tissues from reciprocal crosses between C57Bl6/J and CAST/Ei (BxC and CxB) were collected at 8.5dpc, 17.5dpc, 3dpp and 60dpp. All procedures using animals were reviewed and approved by the Institut Curie Animal Care and Use Committee.

Sequencing details and alignment

The *Dnmt3L-/+* embryos, wildtype embryos and sperm samples were 2x 36bp paired-end sequenced using one lane per sample, except for wildtype embryo sample #1 for which two lanes were used. The liver samples were 12-way multiplexed and 50bp single-end sequenced using 7 lanes. Reads were aligned to the mm9 reference genome using novoalign (http://www.novocraft.com/main/index.php) except for the sperm samples that were aligned using bowtie (Langmead et al., 2009).

DMR identification

DMRs were identified between *1) Dnmt3L-/+* and wildtype embryo samples, and *2)* sperm and wildtype embryo samples, using USeq and DeSeq (Anders and Huber, 2010; Nix et al., 2010). The input alignment data were limited to the unique (default

novoalign/bowtie definitions) and primary alignments of properly paired reads that were not flagged by Picard (http://picard.sourceforge.net/) as potential PCR duplicates and had a minimum mapping quality of 13. Thus, USeq identified DMRs using 32.7M (across 2 samples) versus 41M (2 samples) data points (fragment midpoints) in comparison *1)*, and 86.7M (3 samples) versus 41M (2 samples) data points in *2)*.

DMR annotation

The DMRs determined by USeq in the comparison (*Dnmt3L-/+* versus wildtype embryos) were annotated with the overlapping DMRs from the comparison (sperm versus wildtype embryos), and the parental allele-specific reads counts for the contained SNPs, if any. The read counts were augmented with binomial test results for significant deviation from the expected 1:1 ratio between the parental alleles. Additional third party data were added based on coordinate overlaps and/or maximum distance thresholds using PostgreSQL (http://www.postgresql.org/). These data and their sources are: repeat content (RepeatMasker: http://www.repeatmasker.org/), transcripts (Thierry-Mieg and Thierry-Mieg, 2006), CpG islands (Illingworth et al., 2010), known imprinted gDMRs/ICRs (WAMIDEX: (Schulz et al., 2008), evidence for allele-specific gene expression in mouse brain (Gregg et al., 2010), histone modifications at promoters in mouse embryonic stem cells, fibroblasts and neural progenitor cells (Mikkelsen et al., 2007), MeDIP-chip data comparing mouse parthenogenic and androgenic samples (Hiura et al., 2010), Zfp57/KAP1/SetB1 ChIP-Seq peaks (Quenneville et al., 2011), and average percent methylation of CGIs in wildtype oocytes, *Dnmt3L-/-* oocytes, sperm, blastocysts and mouse ES samples (Kobayashi et al. 2012a).

Allele-specific read count statistics for liver samples

Positional and allele information for approximatively 3.9M SNPs between the C57BL/6 and PWD/PhJ strains was obtained from Mouse Genome Informatics (MGI) at the Jackson laboratories (http://www.informatics.jax.org/). Read pileups over all SNP sites were generated using samtools (Li et al., 2009) and turned into parental allele-specific counts. The observed read counts for the maternal and the paternal alleles of each candidate gDMR (Table 1 and Supplementary Table S1) were tested for consistency with the null hypothesis (H0) of a read being equally likely to originate from either the maternal or the paternal allele (binomial distribution with P=0.5) versus the alternative hypothesis (H1) that the chance of a read coming from the maternal allele is higher (P>0.5). The p-value of the test (not to be confused with P) expresses the chance of making a type 1 error when rejecting H0 in favor of H1. A p-value of 1 was entered in Table 1 and Supplementary Table S1 if there was no informative SNP and hence, no data. Similarly,

instances where read coverage of the SNP was <10 (insufficient power to reject H0 at α=0.05 even for a 9:1 observed skew in the number of reads toward maternal methylation), are highlighted in italics in Table 1.

DNA methylation analysis by MSRE-PCR and bisulfite sequencing

Genomic DNA was isolated by standard procedures, except for sperm, where a modified extraction method was used to eliminate somatic cells present in the epididymus semen (Jeffreys et al., 1994). For MSRE-qPCR, 1μg of DNA was mixed with restriction buffer and then split in two tubes, one corresponding to the digested experiment with 20U of the methylation-sensitive restriction enzyme *McrBC* and twice the amount of recommended GTP, and one to the undigested control with water instead. Samples were incubated at 37°C for 5h, heat inactivated at 65°C for 20 min and 1ng was used for real-time PCR using the SYBR Green technology (Applied Biosystems). For the bisulfite experiment, PCR amplicons were designed to include single nucleotide polymorphisms to infer the parental origin of alleles. The equivalent of at least 300 diploid cells was used per PCR. For oocytes and blastocysts, a method based on agarose bead embedding was used. Methylation analysis of the paternally imprinted *H19* DMR was systematically performed on DNA of pools of oocytes and sperm to verify the lack of somatic contamination (data not shown). For sequencing, we cloned PCR fragments with the pCR2.1 Topo TA cloning kit (Invitrogen). Around 30 clones were analyzed per experiment. All PCR primers are listed in Supplemental Table S2.

ChIP quantitative and allelic analysis

ChIP was performed on chromatin after formaldehyde crosslinking from MEFs according to previously published protocols (Navarro et al., 2005). Details of the antisera used are provided as Supplemental Table S3. Quantitative analysis of immunoprecipitated DNA was determined in real-time PCR assays using the SYBR Green technology (Applied Biosystems). The fold enrichment of each target sequence was calculated as 2 to the power of the cycle threshold (Ct) difference between Input and antibody Bound fractions (% Input), normalized to those of the *Tbx15* housekeeping gene or to IAP sequences. Values given represent the average of three ChIP assays performed on independent chromatin preparations, one from BxC MEFs and two from CxB MEFs.

Allelic analyses were carried out by pyrosequencing with the PyroMark Q24 Pyrosequencer according to the manufacturer’s instructions (Qiagen), after amplification using a HotStart Taq Polymerase (Promega). The relative level of the two parental alleles in each antibody bound fraction was quantified by the PyroMark Q24 1.0.10 Software

(Qiagen) using the allele quantification method. Assay-specific biases were controlled on Input DNA and H3 bound DNA.

RNA expression analysis

Total RNA was extracted from embryos and tissues (Trizol, Invitrogen). Northern blots were hybridized with PCR-generated probes in PerfectHyb buffer (Sigma). RNA integrity and loading were verified by probing the membrane with a β-actin probe (data not shown). For RT-PCR analyses, RNA was DNase-treated (Qiagen) prior to reverse-transcription with random hexamers (Superscript III, Invitrogen). Quantitative analysis of RNA expression was determined in real-time PCR assays using SYBR Green (Applied Biosystems), relative to *Rrm2* and *Arp0* genes as normalizers. Allelic analyses were carried out either by regular sequencing (Big Dye v3.1) or by pyrosequencing with the PyroMark Q24 Pyrosequencer, as described above. Assay-specific biases were controlled on BxC genomic DNA.

Supplemental References

Anders, S., and Huber, W. (2010). Differential expression analysis for sequence count data. Genome Biol *11*, R106.

Hiura, H., Sugawara, A., Ogawa, H., John, R. M., Miyauchi, N., Miyanari, Y., Horiike, T., Li, Y., Yaegashi, N., Sasaki, H., *et al.* (2010). A tripartite paternally methylated region within the Gpr1-Zdbf2 imprinted domain on mouse chromosome 1 identified by meDIP-on-chip. Nucleic Acids Res *38*, 4929-4945.

Jeffreys, A. J., Tamaki, K., MacLeod, A., Monckton, D. G., Neil, D. L., and Armour, J. A. (1994). Complex gene conversion events in germline mutation at human minisatellites. Nat Genet *6*, 136-145.

Langmead, B., Trapnell, C., Pop, M., and Salzberg, S. L. (2009). Ultrafast and memory-efficient alignment of short DNA sequences to the human genome. Genome Biol *10*, R25.

Li, H., Handsaker, B., Wysoker, A., Fennell, T., Ruan, J., Homer, N., Marth, G., Abecasis, G., and Durbin, R. (2009). The Sequence Alignment/Map format and SAMtools. Bioinformatics *25*, 2078-2079.

Navarro, P., Pichard, S., Ciaudo, C., Avner, P., and Rougeulle, C. (2005). Tsix transcription across the Xist gene alters chromatin conformation without affecting Xist transcription: implications for X-chromosome inactivation. Genes Dev *19*, 1474-1484.

Nix, D. A., Di Sera, T. L., Dalley, B. K., Milash, B. A., Cundick, R. M., Quinn, K. S., and Courdy, S. J. (2010). Next generation tools for genomic data generation, distribution, and visualization. BMC Bioinformatics *11*, 455.

Thierry-Mieg, D., and Thierry-Mieg, J. (2006). AceView: a comprehensive cDNA-supported gene and transcripts annotation. Genome Biol *7 Suppl 1*, S12 11-14.
